# A Systematically Improved High Quality Genome and Transcriptome of the Human Blood Fluke *Schistosoma mansoni*

**DOI:** 10.1371/journal.pntd.0001455

**Published:** 2012-01-10

**Authors:** Anna V. Protasio, Isheng J. Tsai, Anne Babbage, Sarah Nichol, Martin Hunt, Martin A. Aslett, Nishadi De Silva, Giles S. Velarde, Tim J. C. Anderson, Richard C. Clark, Claire Davidson, Gary P. Dillon, Nancy E. Holroyd, Philip T. LoVerde, Christine Lloyd, Jacquelline McQuillan, Guilherme Oliveira, Thomas D. Otto, Sophia J. Parker-Manuel, Michael A. Quail, R. Alan Wilson, Adhemar Zerlotini, David W. Dunne, Matthew Berriman

**Affiliations:** 1 Wellcome Trust Sanger Institute, Wellcome Trust Genome Campus, Hinxton, United Kingdom; 2 Texas Biomedical Research Institute, San Antonio, Texas, United States of America; 3 Departments of Biochemistry and Pathology, University of Texas Health Science Center, San Antonio, Texas, United States of America; 4 Center for Excellence in Bioinformatics, Centro de Pesquisas René Rachou, Fundação Oswaldo Cruz, Belo Horizonte, Minas Gerais, Brazil; 5 Genomics and Computational Biology Group, Centro de Pesquisas René Rachou, Fundação Oswaldo Cruz, Belo Horizonte, Minas Gerais, Brazil; 6 National Institute for Science and Technology in Tropical Diseases, Centro de Pesquisas René Rachou, Fundação Oswaldo Cruz, Belo Horizonte, Minas Gerais, Brazil; 7 Department of Biology, University of York, Heslington, York, United Kingdom; 8 Department of Pathology, University of Cambridge, Cambridge, United Kingdom; IBERS, United Kingdom

## Abstract

Schistosomiasis is one of the most prevalent parasitic diseases, affecting millions of people in developing countries. Amongst the human-infective species, *Schistosoma mansoni* is also the most commonly used in the laboratory and here we present the systematic improvement of its draft genome. We used Sanger capillary and deep-coverage Illumina sequencing from clonal worms to upgrade the highly fragmented draft 380 Mb genome to one with only 885 scaffolds and more than 81% of the bases organised into chromosomes. We have also used transcriptome sequencing (RNA-seq) from four time points in the parasite's life cycle to refine gene predictions and profile their expression. More than 45% of predicted genes have been extensively modified and the total number has been reduced from 11,807 to 10,852. Using the new version of the genome, we identified *trans*-splicing events occurring in at least 11% of genes and identified clear cases where it is used to resolve polycistronic transcripts. We have produced a high-resolution map of temporal changes in expression for 9,535 genes, covering an unprecedented dynamic range for this organism. All of these data have been consolidated into a searchable format within the GeneDB (www.genedb.org) and SchistoDB (www.schistodb.net) databases. With further transcriptional profiling and genome sequencing increasingly accessible, the upgraded genome will form a fundamental dataset to underpin further advances in schistosome research.

## Author Summary

Schistosomiasis is a disease caused by parasitic blood flukes of the genus *Schistosoma*. Human-infective species are prevalent in developing countries, where they represent a major disease burden as well as an impediment to socioeconomic development. In addition to its clinical relevance, *Schistosoma mansoni* is the species most widely used for laboratory experimentation. In 2009, the first draft of the *S. mansoni* and *S. japonicum* genomes were published. Both genome sequences represented a great step forward for schistosome research, but their highly fragmented nature compromised the quality of potential downstream analyses. In this study, we have substantially improved both the genome and the transcriptome resources for *S. mansoni*. We collated existing data and added deep DNA sequence data from clonal worms and RNA sequence data from four key time points in the life cycle of the parasite. We were able to identify transcribed regions to single-base resolution and have profiled gene expression from the free-living larvae to the early human parasitic stage. We uncovered extensive use of single transcripts from multiple genes, which the organism subsequently resolves by *trans*-splicing. All data from this study comprise a major new release of the genome, which is publicly and easily accessible.

## Introduction

*Schistosoma* spp. are platyhelminth (flatworm) parasites responsible for schistosomiasis, a tropical disease endemic in sub-tropical regions of Africa, Brazil, Central America, regions of China and Southeast Asia, which causes serious morbidity, mortality and economic loss. An estimated 779 million people are at risk of infection and more than 200 million are infected [1].

The paired adult males and females of *S. mansoni* reside in the hepatic portal vasculature, each female depositing 200–300 eggs per day near the intestinal wall. These eggs either pass into the gut lumen to be voided in the faeces and continue the life cycle or pass up the mesenteric veins and lodge in the liver, where they can cause serious pathology including granulomatous inflammation response and fibrosis. On contact with fresh water, free-living motile miracidia hatch from the eggs to infect aquatic snails (*Biomphalaria* spp.), where parasites undergo two rounds of asexual multiplication and are released as infective cercariae into water. Cercariae infect the human host, by penetrating unbroken skin, and transform into schistosomula. After several days the parasites exit the cutaneous tissue via blood (or lymphatic) vessels and travel first to the lungs and onward into the systemic vasculature. They may make multiple circuits before arriving in the hepatic portal system; only then do they start to feed on blood, mature and pair up, the whole process taking approximately five weeks [2].

Two *Schistosoma* draft genomes (*S. mansoni* and *S. japonicum*) were recently published [3], [4] and represent the only described genomes amongst parasitic flatworms to date. Their assemblies were generated by conventional capillary sequencing but are highly fragmented (*S. mansoni*, 19,022 scaffolds; *S. japonicum*, 25,048 scaffolds) and severely compromise gene prediction, as well as comparative and functional genomics analyses. The transcriptome has similarly only been partially characterised by large-scale expressed sequence tag (EST) sequencing and low-resolution cDNA-based microarrays.

Second-generation sequencing technologies provide new opportunities to characterise both genomes and transcriptomes in depth. In addition to whole genome *de novo* sequencing [5], [6] and genome improvement [7], massively parallel cDNA sequencing (RNA-seq) can identify transcriptionally active regions at base-pair resolution [8]–[11] and accurately define the exon coordinates of genes [12]. In addition, the quantitative nature and high dynamic range of RNA-seq allows gene expression to be scrutinised [11], [13], [14] in a more sensitive and accurate way than other previous high-throughput methods [15], [16].

In this study we systematically improved the draft genome of *S. mansoni*, using a combination of traditional Sanger capillary sequencing, second generation DNA sequencing from clonal parasites and reanalysis of existing genetic markers [17]. This allowed us to assemble 81% of the genome sequence into chromosomes. We have also used RNA-seq data from several life-cycle stages to refine the structures of 45% of existing genes as well as to identify new genes and alternatively spliced transcripts. In addition to *cis* splicing, our data highlight extensive *trans*-splicing and provide clear evidence that the latter can be used to resolve polycistronic transcripts. With RNA-seq we profiled the parasite's transcriptome during its transformation from the free-living, human-infectious cercariae to the early stages of infection and in the mature adult. As the infective form transforms into a mammalian-adapted parasite, the relative abundance of transcripts shifts markedly during a 24-hour period, from those involved in glycolysis, translation and transcription to those required for complex developmental and signalling pathways.

The improved sequence and new transcriptome data are available to the community in a user-friendly and easy to query format via both the GeneDB (www.genedb.org) and SchistoDB (www.schistodb.net) databases. These data demonstrate that revisiting a previously published draft genome, to upgrade its quality, is an option that should not just be reserved for model organisms.

## Materials and Methods

The full description of materials and methods is presented in Supplementary Materials (Text S1). A synopsis of the methods used in this paper is presented below.

### Parasite material, library preparation and sequencing

*S. mansoni* clonal DNA was obtained from single miracidium infections of *Biomphalaria* snails. Male and female adults (NMRI strain, Puerto Rican origin) were obtained from infected C57Bl/6 mice. DNA extraction was performed and sequencing libraries were prepared as previously described [18]. Eight and lanes were sequenced for the male samples and one lane for the female sample, both as 108-base paired reads. For RNA-seq samples, total RNA samples were obtained from cercariae, 3 hours and 24 hours post-infection schistosomula, and 7-week old mixed sex adult worms. Schistosomula samples were obtained using mechanical transformation [19]. RNA-seq libraries were prepared using a modified version of the protocol described in [8] and sequenced as 76-base paired reads. All samples were sequenced using the Illumina Genome Analyzer IIx platform. Raw sequence data were submitted to public data repositories; DNA reads were submitted to ENA http://www.ebi.ac.uk/ena/ under accession number ERP000385 and RNA-seq reads were submitted to ArrayExpress http://www.ebi.ac.uk/arrayexpress/ under accession number E-MTAB-451).

### Generating a new assembly and transferring previous gene annotation

The Arachne assembler (version 3.2, [20]) was used to produce a new assembly using the existing capillary reads from the previously published draft assembly [3], supplemented with an additional ∼90,000 fosmid and BAC end sequences. FISH-mapped BACs [3] were also end-sequenced generating 438 reads that were incorporated into the assembly. Illumina reads were used to close gaps with the IMAGE pipeline [7]. The sequences of 243 published linkage markers [17] of *S. mansoni* were retrieved and used as anchors within the assembly by incorporating them as *faux* capillary reads. Scaffolds containing these reads were ordered, orientated and merged into chromosomes. Except where indicated, all analyses reported in the present study refer to a frozen dataset taken at this stage of the assembly process (*S. mansoni* genome v5.0, available at http://www.sanger.ac.uk/resources/downloads/helminths/schistosoma-mansoni.html). All comparisons were made against the previously published draft genome (v4.0).

As part of the active finishing process, we randomly checked ∼20% (2,062) of the gaps automatically closed by IMAGE and found 90% of these could be verified by visual inspection. Contigs containing telomeric repeat sequences (TTAGGG) [21] were extended by oligo-walking pUC clones until a unique sequence was identified. Where the unique sequence was linked to a known marker, the telomere could be placed onto a chromosome. All manual improvement changes were included in a subsequent snapshot of the data (v6.0).

To transfer the existing annotation to the latest reference we used RATT [22] (with the old assembly split into four parts and using options –q and –r) to define regions with synteny between both assemblies and transform the annotation coordinates onto the new assembly.

The annotated genome sequence was submitted to EMBL http://www.ebi.ac.uk/embl/ under the accession numbers HE601624 to HE601631 (nuclear chromosomes); HE601612 (mitochondrial genome); and CABG01000001 to CABG01000876 (unassigned scaffolds).

### Gene finding using RNA-seq

Each lane of RNA-seq reads was independently aligned to the genome using TopHat [23] and the resulting alignment files used as the input for the gene finder Cufflinks [12]. Transcript fragments with less than 10× average read depth coverage and fewer than 50 codons were excluded from subsequent analyses. JIGSAW [24] was used to combine existing models with Cufflinks' transcript fragments. The final set of gene models can be accessed through GeneDB http://www.genedb.org/Homepage/Smansoni and SchistoDB http://www.schistodb.net.

### *Trans*-splicing and polycistronic transcription

RNA-seq read pairs that contained the splice leader (SL) sequence [25] were used to find *trans*-splicing sites; where a gene was found within 500 bases from a *trans*-splice site its transcript was tagged as putative *trans*-spliced. By looking for genes whose 3′ end was located within 2,000 bp upstream of a putative *trans*-spliced acceptor site, putative polycistronic units were identified. RT-qPCR was performed to validate both *trans*-spliced and polycistronic transcripts.

### Quantification of RNA-seq and differential expression

RNA-seq reads were aligned to the reference genome using SSAHA2 [26]. A minimum mapping score 10 was applied to filter aligned reads. Reads per gene and RPKMs (reads per Kilobase per million mapped reads [8]) were calculated using only coding regions coordinates. We also estimated the background signal for non-coding regions (RPKM<2). Total reads per gene were used to identify differentially expressed genes (using only genes with >background RPKM) in pair wise comparisons (adjusted p-value<0.01 – adjusted for multiple testing [27]) using the edgeR package [28] implemented in the Bioconductor R-package [29]. Gene Ontology (GO) term enrichment analysis was performed with TopGO [30], also implemented in R [31].

### Ethics statement

The procedures involving animals in the UK were carried out in accordance with the UK Animals (Scientific Procedures) Act 1986, and authorised on personal and project licences issued by the UK Home Office. The study protocol was approved by the Biology Department Ethical Review Committee at the University of York. The procedures involving animals in the US were carried out in strict accordance with the Animal Welfare Act (Public Law 91–579) and the recommendations in the Guide for the Care and Use of Laboratory Animals of the National Institutes of Health (OLAW/NIH, 2002). The protocol was approved by the University of Texas Health Science Center Institutional Animal Care and Use Committee (IACUC, Protocol Number: 08039x).

## Results

### An improved chromosomal assembly

Using the existing Sanger-sequencing data from the published draft genome [3], supplemented with an additional ∼90,000 fosmid and BAC ends, we produced an improved version of the *S. mansoni* genome *de novo* using the Arachne assembler [20]. With only 885 scaffolds, the new assembly contains less than one-twentieth of the original number of scaffolds (Table 1). Half of the 364.5 Mb genome is represented in scaffolds greater than 2 Mb and 90% are over 0.5 Mb. Ordering and orientating scaffolds based on 243 available linkage markers [17] and end-sequences from FISH-mapped BACs [3] further improved the continuity of the genome. The largest scaffold of 10 Mb contains 8 microsatellite markers from Chromosome 6 and no ambiguities, i.e., the order of the contigs in this scaffold is the same as the order of the markers in the linkage group. Chromosome 1 represents the largest placed chromosome of 79.6 Mb with 41.8 Mb of the sequences ordered and concatenated as a single scaffold. There were only 6 microsatellite ambiguities in the whole assembly and these were corrected by targeted manual finishing.

**Table 1:** Characteristics of the old and improved Schistosoma mansoni genome assemblies.

	Old version^a^	New version^b^
Assembly size (Mb)	374.9	364.5
Proportion assigned to chromosome (%)	55	81
*Contig statistics*		
Number	50,292	9203
Average length (kb)	7.5	39.4
N50 length (kb)	16.3	78.3
Largest contig (kb)	139.4	460
*Scaffold statistics*		
Number	19,022	885
Average length (kb)	20	411.9
N50 length (Mb)	0.8	32.1
Largest scaffold (Mb)	4.2	65.5^c^
*Accuracy assessment using mapped reads* d		
Properly mapped read pairs^e^ (%)	91.5	91.8
Pairs mapped too far apart^f^ (%)	0.23	0.18
Pairs mapped in wrong orientation (%)	0.28	0.27
Pairs mapped to different scaffolds (%)	0.17	0.11
Only one mate mapped (%)	2.44	2.47
Unmapped (%)	5.37	5.16

a Version 4.0 of the *S. mansoni* genome was the published draft genome [3]. Note the sequence in version v4.0 was identical to the previously released v3.1.

b Version 5.0.

c This figure refers to a “super-scaffold” of chromosome I sequence where 41 assembly-scaffolds are linked using genetic markers.

d 101,079,940 pairs of Illumina reads.

e Reads mapped in their correct orientation and at a distance apart that corresponds to that predicted by the fragment library size.

f Reads that mapped in their correct orientation but at a distance apart that did not match that predicted by the fragment library size.

**Figure 1:**
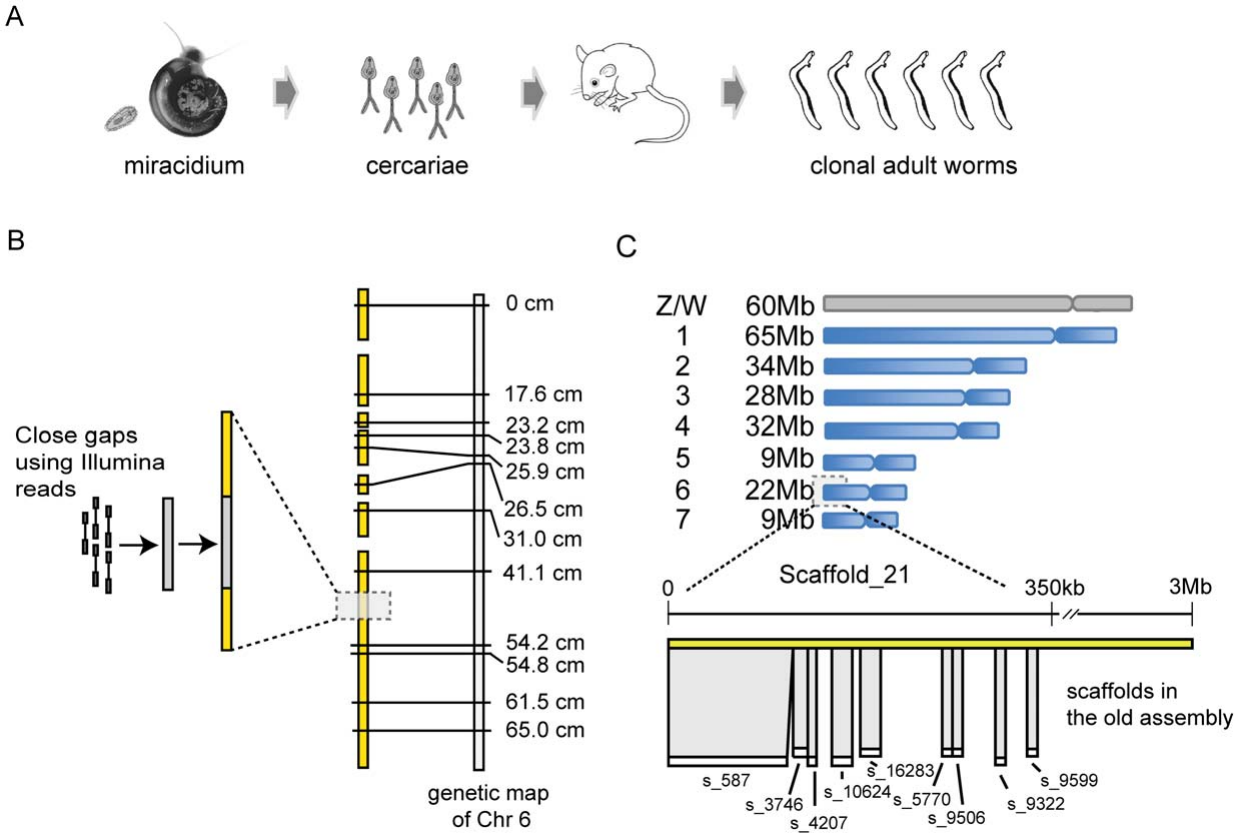
Improving the genome assembly of S. mansoni. (A) Generation of clonal adult worms for Illumina sequencing. A single *B. glabrata* snail was infected with one miracidum only. The normal asexual reproduction stage of the sporocyst in the snail produces thousands of clonal cercariae that were used to infect mice. Clonal adult worms were recovered 7 weeks post-infection and processed for DNA extraction. (B) Closing gaps with IMAGE. Illumina data generated from the clonal adult worms were used to close gaps in the assembly using IMAGE [7] and, in conjunction with previous sequencing data, linkage markers and BAC ends, allowed the genome to be assembled into chromosomes. (C) Organisation of the *S. mansoni* genome into chromosomes. Top: The total length of the scaffolds that have evidence (either linkage markers or FISH-mapped BACs) assigning them to the 7 autosomal and W/Z chromosomes. Bottom: A schematic diagram showing the example of supercontig_21 (3 Mb), which was allocated to chromosome 6 using information from genetic mapping [17], and was able to link together 9 supercontigs from the old assembly into the first 350 kb.

We then used genomic DNA from a clonal adult male population (see Methods, Text S1 and Figure 1A) to reduce the number of gaps within scaffolds and generally improve the assembly. Using the Genome Analyzer IIx platform, we generated 11 Gb of 108-base paired reads, approximately 60-fold genome coverage. IMAGE [7] was then used to iteratively extend contigs into gaps by performing local assemblies of the Illumina reads (Figure 1B). After 33 iterations with a range of *k*-mer sizes, IMAGE closed a total of 11,158 gaps (53.4% of all possible gaps). The closed gaps had an average length of 315 bp with the largest gap being 6.5 kb (Figure S1). The statistics of the improved new assembly are shown in Table 1. Compared with the previous draft assembly, the number of contigs was reduced from 50,292 to 9,203 and the N50 was increased from 16 to 78 kb.

Because the linkage markers were associated with much larger scaffolds, we were able to allocate an additional 84 Mb of consensus sequence data into individual chromosomes, bringing the total to 81% (Figure S2). The improvement is best illustrated in chromosome 6, which consists of the largest and 5 smaller scaffolds in the new assembly, but corresponds to 1,537 scaffolds from the old assembly. Illumina reads from clonal worms, mapped to both assemblies, were also used to assess assembly improvement. Table 1 shows that the mapping statistics were broadly similar in both assemblies. However, in terms of absolute numbers, more reads mapped to the new assembly despite the total genome length having been reduced by ∼10 Mb. Further, an increased number of read-pairs mapped in their correct orientation, within a distance predicted by the sequencing library fragment size, indicating fewer mis-assemblies.

Following assembly, the genome was further improved by manual finishing. In particular, 305,465 Sanger reads (comprising repetitive sequences that were previously excluded by assembly software) were manually incorporated, three more scaffolds were ordered into chromosome sequences, and 17 new contigs were assembled to further extend the ends of chromosomes. For example, by closing 33 gaps, one end of chromosome 6 has been extended by 1.4 Mb and now includes its telomeric tract.

### Sex chromosomes share significant sequence

The *S. mansoni* genome has one pair of sex chromosomes. Females are the heterogametic sex with both Z and W chromosomes and males are homogametic with a ZZ pair. We found Z and W assembled together into 34 scaffolds, which could be ordered and orientated based on 51 previously reported genetic linkage markers [17] and comprised a total of 59 Mb. We used differences in coverage of reads mapped from male and female DNA, to identify both Z and W specific regions (Text S1). Approximately 30% of the Z/W chromosome was Z-specific (Figure S3) and contained 23 Z-specific genetic markers [17]. Amongst the unplaced sequences that lack genetic markers, were an additional 69 Z-specific scaffolds (>100 kb) and a further 114 unplaced scaffolds (∼1.1 Mb) that were W-specific. Repeats comprise 90% of the latter, and include previously identified female-specific repeat [32] as well as 0.1 Mb of previously uncharacterised female-specific sequences. These scaffolds usually have female reads mapped many fold higher than the average coverage of the assembly, for example scaffold 1570 has 26 times higher coverage than the average, suggesting that the heterochromatin portion of the W chromosome have been collapsed into these scaffolds. Based on the differences between the genome-wide assembly coverage and the coverage of these scaffolds, we estimate these heterochromatin portions of the W chromosome to comprise ∼3.3 Mb collapsed into the 1 Mb of consensus. Interestingly, the W-specific scaffolds appear to contain no coding genes whereas the Z-specific portion of Z/W sequence contains 782 genes, ∼95% of which exist as single-copies within the assembly.

### The mitochondrial genome

Amongst the unassembled reads there were 5,647 that originated from mitochondrial DNA. An independent assembly of these reads using CAP3 [33] generated a single contig of 21 kb (to which 15 scaffolds from the previous genome assembly could be aligned). The first 14 kb of the contig was 99.9% identical to the published coding portion of the *S. mansoni* mitochondrial genome [34]. Based on restriction fragment analysis, a long non-coding region that is repetitive and highly variable between individuals has previously been partially characterised [35]. In our data, the additional 9 kb non-coding portion of the mitochondria genome is now complete and comprises known 62 bp repeats [35], plus additional 558 bp repeats and long tracts of low complexity sequence.

### Improvements to gene models using RNA-seq

We obtained total RNA from four time points of the life cycle of *S. mansoni*: 1) free-living mammalian-infectious cercariae, mechanically transformed schistosomula at 2) three hours and 3) twenty hours post infection, and 4) seven-week old mixed-sex adults recovered from hamster host. The 183 million 76-base RNA-seq read pairs were mapped to the new reference genome using SSAHA2 alignment tool [26]. An average 70% of the RNA-seq reads generated in each sequenced library aligned as proper pairs to the genome (Table 2), an improvement over the previous version of the genome. Less than 6% of reads mapped to the mitochondrial genome in each sample; the lowest (0.5%) corresponding to the schistosomula stages.

**Table 2:** Summary of RNA-seq mapping.

	Cerc	3 h Som	24 h Som	Adult
**Total read pairs sequenced (out of 183,590,080)**	69,498,003	53,041,873	50,528,949	10,521,255
Properly mapped read pairs^a^ (%)	70.7	68.6	69.8	72.3
Additional properly mapped read pairs in new assembly^b^ (%)	2.0	0.2	0.4	2.8
Pairs mapped to repeats (%)	23.8	14.0	16.2	19.7
Pairs mapped to different scaffolds (%)	0.2	2.1	3.0	0.3
One mate mapped or mapped in wrong orientation (%)	4.3	12.2	9.7	6.1
Unmapped (%)	1.0	3.2	1.4	1.6
**Proportion of reads mapped to mitochondria**	5.1	0.6	0.4	3.7

Number of RNA-seq reads mapped using SSAHA2 to the genome from libraries prepared from cercariae (Cerc); 3-hour post-infection schistosomula (3 h Som); 24-hour post-infection schistosomula (24 h Som); and mixed male and female adult worms (Adult).

a reads mapped within expected distance apart and in the correct orientation.

b reads that were properly mapped to the new assembly but not in the previous.

**Figure 2:**
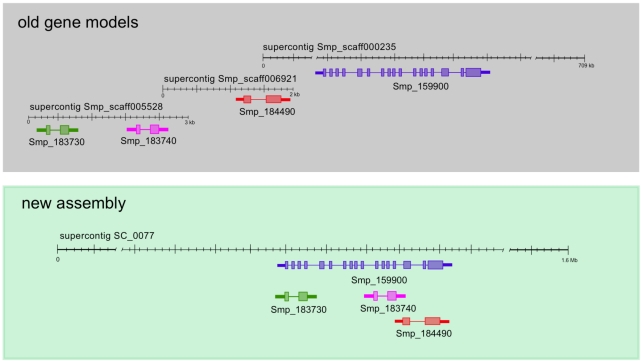
Removal of assembly redundancies produces a more reliable set of gene models. Gene models were migrated from previous version using RATT [22]. Repeats and sequencing errors in the old assembly resulted in ambiguities and sequences being represented more than once. In the new version, many scaffolds coalesced into one region and hence the gene models contained in them overlap each other. In this example, four supercontigs from the previous version collapsed on an unplaced region of Chromosome 3 in the new assembly. The smaller gene models are now obsolete as they were clearly incomplete annotations and their coding region are part of the exons of the larger gene model.

The majority (91%) of the 11,799 gene models from the previous version of the genome could unambiguously be transferred onto the new assembly. Splitting gene models from the previous assembly increased the gene count by 307; however, the coalescence of genes previously located on multiple different scaffolds caused some redundancy (an example is shown in Figure 2), removal of which reduced the number of transferred genes to 10,123. Of the 1,065 genes that could not be transferred to the new assembly, at least 83% were presumed to represent incorrect annotations due to a lack of sequence similarity and their short lengths, 1- or 2-exon structures (Figure S4) or a lack of start or stop codons.

RNA-seq data has been used to refine and improve gene model predictions in various organisms [10], [36], [37]. In the first draft of the *S. mansoni* genome, gene models were generated using a combination of *ab initio* gene predictions and EST evidence [38], with only a few hundred manually curated genes. To systematically upgrade the quality of annotations, we aligned pooled RNA-seq reads using TopHat [23], which allows gaps in the read-to-reference alignment at putative splice sites. Using the upgraded genome sequence 30% more RNA-seq reads with putative splice junctions aligned, highlighting putative new genes or structural refinements that could be made to existing genes.

Cufflinks [12] was used to aid the refinement of gene structures by creating transcript “fragments” with sharply defined exon boundaries [23]. Using transcript fragments with at least 10 reads coverage at each base we found 78% of previous gene models had evidence of transcriptional activity within the sampled life cycle stages. Of these models, 3,604 (45%) were modified to include new exons derived from RNA-seq data, hence generating alternative gene predictions (Table 3). Using the transcript data as a guide, 236 genes were merged and 26 split into two or more gene models.

**Table 3:** Fate of gene models.

	Number
*Total gene models in old genome version* a	**11,719**
Not transferred	1,088
Deleted models	545
Split or merged models	731
Models with additional exons	3,438
Models that have been automatically replaced	1,116
New genes	504
*Genes in new version* b	**10,852**

The criteria for including genes into each category are described in the main text.

a Version 4.0.

b Version 5.0.

**Figure 3:**
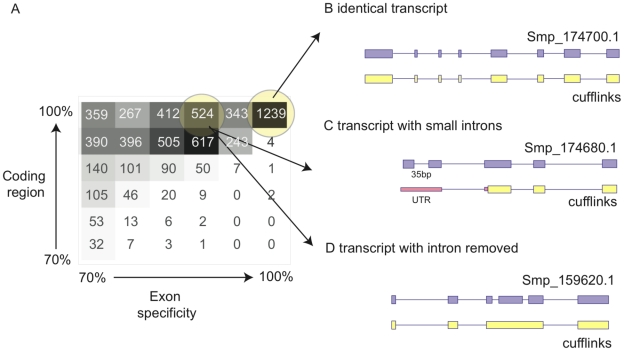
Improvement of gene annotation using RNA-seq. (A) Heatmap displaying comparisons between previous gene models and transcript fragments generated from Cufflinks. For each model, the extent of coding region that overlaps with a Cufflinks' model and the proportion of correctly predicted exon boundaries was calculated and categorised into bins of 70–100%. Models in this plot were excluded with less than 70% of their exon boundaries or coding regions predicted. (B), (C) and (D) Example scenarios of Cufflinks' models compared with previous gene models where (B) the Cufflinks prediction is identical to the 1,239 existing models; (C) Cufflinks fails to identify small introns; (D) Cufflinks removes incorrect introns present in the previous gene model, probably due to the improved assembly which, by correcting gaps, produced a longer single exon while the reading frame is preserved.

To assess the accuracy of gene models, we calculated two metrics: the proportion of intron-exon junctions found in previous models that matched to the same intron-exon junction in a transcript fragment, and the proportion of the coding sequence in previous models that overlapped with the transcript fragments. Figure 3A is a heatmap showing these two metrics; existing models are clustered around top right of the plot, which indicates that RNA-seq evidence-based transcript fragments are similar to the existing models. Sixteen percent of gene models were perfectly reproduced by the transcript fragments (Figure 3B), while 90% of gene models with transcriptional evidence have at least 70% of the coding region covered by the transcript fragments.

In the new dataset, only 53% of gene models have at least 70% of their exon boundaries preserved. There are two main reasons for this low specificity in predicting exon boundaries. First, Cufflinks was unable to successfully predict the small introns typically observed in the 5′ end of many *S. mansoni* genes (Figure 3C and [3]). Consistently, when the first four exons of the old gene models were excluded, we found that transcript fragments could perfectly predict 90% of exon boundaries. Second, sequencing errors in the previous assembly resulted in introns being falsely incorporated into gene models during prediction to compensate for apparent frameshifts. These “intron” sequences are no longer necessary to preserve the reading frame and were identified as part of exons by Cufflinks in the new assembly (Figure 3D). For the two reasons above, we used JIGSAW [39] to combine existing models with those produced from RNA-seq data, resulting in 1,264 exon coordinates being changed.

We identified 1,370 transcripts corresponding to putative full length coding sequences but which did not overlap with existing gene models. To check whether they indeed represented novel genes, we first screened them against known repeats and transposable elements. The 36 previously published transposable element sequences in *S. mansoni* matched 866 of the transcribed fragments, the longest of which (5,061 bp) was 99% identical to the coding portion of the LTR retrotransposon Saci-1 [40].

Of the remaining 504 complete transcript fragments we found sequence similarity for 231 in the NCBI nr protein database, mostly to other genes already annotated in *S. mansoni* (presumably representing gene duplications or members of multi-gene families) or *S. japonicum*. However, seven out of the remaining 273 full-length transcript fragments did show at least one conserved domain: a putative Tpx-1/SCP related allergen, a rhodopsin-like GPCR domain, a DNA-protein interaction domain, a epidermal growth factor-like (EGF-like) domain, and a polypeptide encoding a fascicline-like domain (FAS1) domain), and two transcripts with ArsR transcriptional regulator sequences. The new transcript fragments were on average shorter (261 bp) and exhibited unusual codon usage (Wilcoxon rank sum test, p<0.01, Figure S5) compared with a typical schistosome gene. Although we cannot rule out at this stage that the small set of atypical genes are non-coding RNA species, they are included in the total number of putative protein coding genes, which stands at 10,852.

### *Trans*-splicing

**Figure 4:**
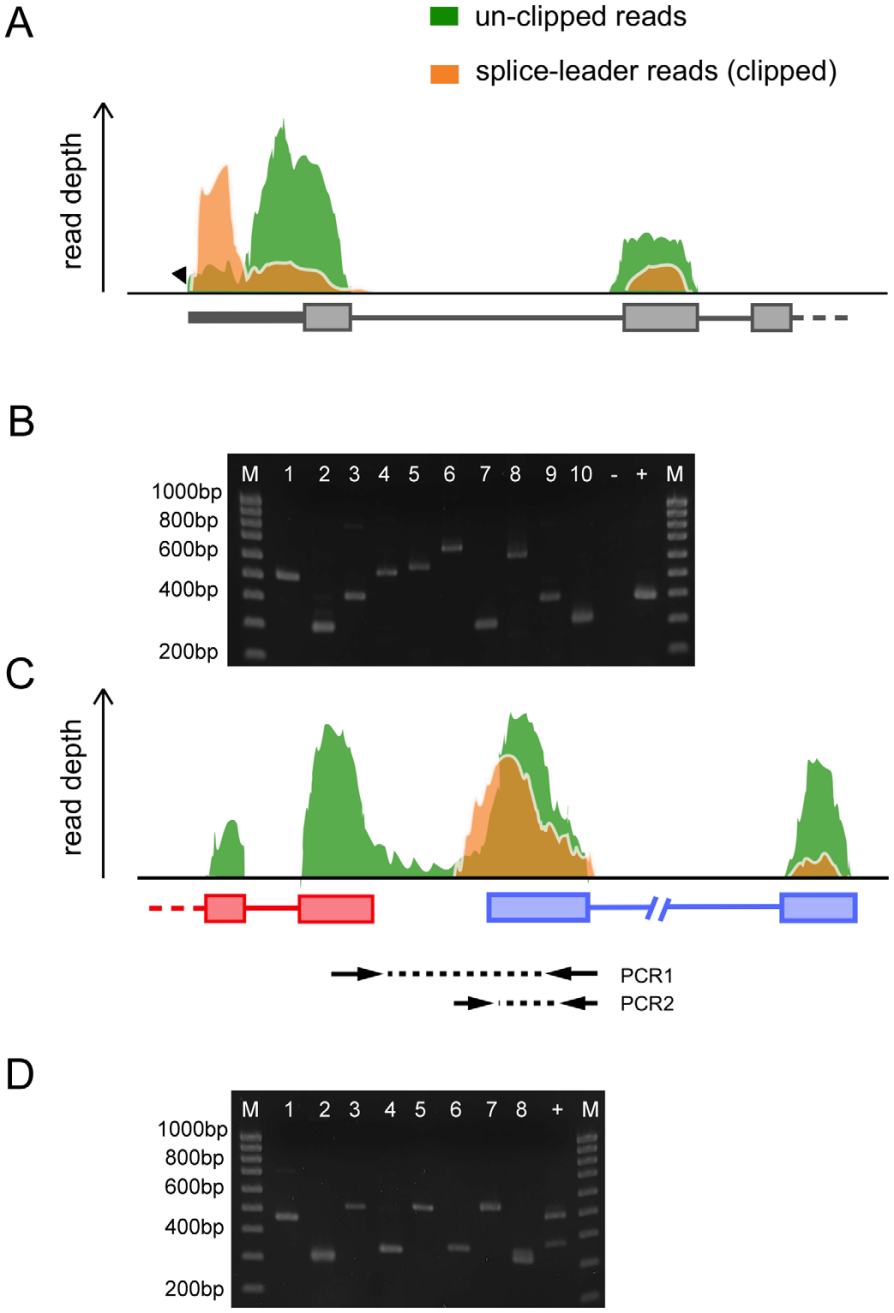
RNA-seq reveals trans-spliced transcripts. (A) Schematic view of the 5′ end of *trans*-spliced gene Smp_176420. Shaded coverage plots represent non-normalized RNA-seq reads still containing the spliced-leader (SL) sequence (green – unclipped reads) and reads previously found to contain the SL sequence (orange - clipped). In the latter, the SL sequence was removed prior to aligning the reads to the genome; which improved the reads mapability (lower in the unclipped reads than in the orange reads). (B) RT-PCR validation of 10 putative trans-spliced genes with SL1 as forward primer and a gene-specific reverse primer. Smp_024110.1, previously described as trans-spliced [41], was included as a positive control (indicated with ‘+’) while Smp_045200.1 was included as a negative control (‘−’). All PCRs but one (Smp_176590.1) show bands corresponding to expected PCR product size. (C) Schematic view of the putative polycistron Smp_079750-Smp_079760. PCR1 represents the amplicon obtained from the unprocessed polycistronic transcript containing the intergenic region while PCR2 the trans-spliced form of Smp_079760. (D) RT-PCR validation of 5 putative polycistrons and a positive control (Smp_024110-Smp_024120; lane 9) previously reported in [45]. Each putative polycistron was subjected to two PCRs that correspond to PCR1 (e.g lane 1) and PCR2 (e.g lane 2) in panel C.

Both *cis* and *trans*-splicing are used to produce mature transcripts in *S. mansoni*. By filtering for RNA-seq reads containing the spliced leader (SL) sequence [25], strongly supported *trans*-splicing events could be mapped on a genome-wide scale and highlighted 1,178 (∼11%) genes (an example is shown in Figure 4A), a figure in close agreement with a previous prediction [41]. For validation, we randomly chose ten putative *trans*-spliced gene models and could verify the existence of their *trans*-spliced transcripts by RT-PCR (Figure 4B, Table S1). In many cases, mapping information suggests a second *trans*-splicing acceptor site, usually within 20–50 bases from the primary acceptor site, indicating that alternative splicing operates at the *trans* as well as *cis* levels. Using Gene Ontology enrichment [30], we could find no particular functions or processes enriched within the *trans*-spliced genes, agreeing with the previous report [41].

Polycistronic transcripts originate from a single promoter but are later processed to generate two or more individual mRNAs. This type of transcriptional regulation is characteristic of trypanosomatids [42] and is present in *C. elegans* [43] and other organisms [44]. It has been suggested [45] that the *S. mansoni* Ubiquinol-cytochrome-c-reductase (UbCRBP) and phosphopyruvate hydratase (Smp_024120 and Smp_024110 respectively) genes might be transcribed as a polycistronic unit and that *trans*-splicing of the phosphopyruvate hydratase might resolve the polycistron into individual transcripts. In our study we provide strong evidence that this is indeed the case. One of the characteristics of polycistronic transcripts is a short intergenic distance (<200 bp) between individual “monocistrons”. We identified a total of 46 *trans*-splicing acceptor sites that fall between gene models that have a maximum intergenic distance of 200 bp, and 115 cases (Figure 4C, Table S2) where the intergenic regions expands up to 2 kb (maximum reported for *C. elegans*). We validated four of these polycistrons by RT-PCR (Figure 4D, Table S1) and Sanger sequencing (data not shown). Unlike *C. elegans*, which uses a second spliced leader (SL2) to resolve polycistrons [43], *S. mansoni* seems to use the same SL for both polycistronic- and non-polycistronic- *trans*-spliced transcripts. The role of polycistrons in schistosome gene expression remains to be determined but no pattern could be discerned between the ascribed functions of genes within each polycistron.

### Transcriptome analysis and differentially expressed genes

In order to profile the transcriptional landscape of the parasite establishing in the mammalian host, the RNA-seq data from four key time points in the parasite's life cycle were analysed independently. Consistent with RNA-seq experiments elsewhere [16], we found good reproducibility between biological replicates, indicated by high correlation coefficients (average Pearson correlation of log RPKM values, across five pairs of biological replicates, was 0.95; Figure S6).

A total of 9,535 (88%) genes were expressed (above an empirically determined background RPKM cut-off of 2 – Text S1 and Figure S7) in at least one surveyed time point and the remaining 12% were regarded as genes with expression too low to be detected or expressed during life stages not surveyed in this study (e.g. intra-molluscan stages) and therefore were excluded from further analysis. Of the excluded genes, 65% are annotated as hypothetical proteins (higher than the genome-wide figure of 44%).

**Figure 5:**
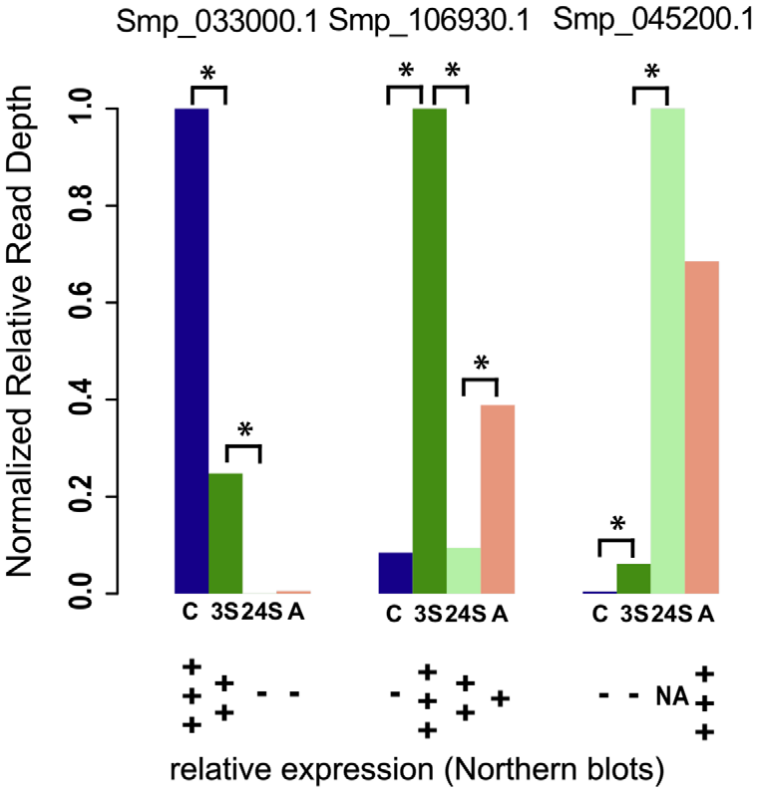
Comparison of expression of genes previously identified to be developmentally regulated. Barplots represent relative normalized reads (from RNA-seq data) for 3 transcripts, asterisks represent comparisons where differential expression is significant (adjusted p-value<0.01). Relative expression reported in the literature [46], [49], [51] is shown at the bottom (+++, high expression, ++ medium expression, + some expression, − not expressed, NA no information available). C = cercariae, 3S = 3-hour schistosomula, 24S = 24-hour schistosomula, A = adult.

To gain better insight into the resolution of the RNA-seq approach in *S. mansoni*, we compared our results with a few example genes that have been described to undergo pronounce changes in their expression along the parasite's life cycle: an 8 kDa calcium binding protein, associated with tegument remodelling during cercariae transformation into schistosomula [46], [47]; a heat shock protein 70 (HSP70), active in schistosomula after penetration through mammalian host skin [48]–[50]; and the tegument antigen Sm22.6 [51], associated with resistance to re-infection in adult patients of endemic areas [52]. Our RNA-seq results broadly agree (Figure 5) with relative gene expression measurements obtained through other approaches. We also investigated how well the RNA-seq data correlate with previous microarray studies [53], [54]. Comparing normalised intensity values of the array features against the RNA-seq read depth for each microarray probe location in the genome (Figure S8) suggests that these data broadly correlate (Pearson's correlation of the log values 0.67).

**Figure 6:**
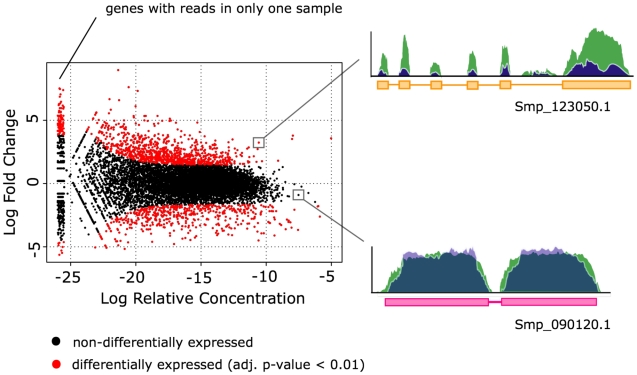
Detection of differentially expressed genes. The plot (left) shows the log fold change (y-axis) vs. log relative concentration (x-axis) for the cercariae – 3-hour schistosomula comparison. A total of 1,518 genes are differentially expressed between these two life cycles stages (adjusted p-value<0.01). On the right, example coverage plots for differentially and non-differentially expressed genes. Of particular interest, genes up regulated in the 3-hour schistosomula stage are enriched in G-protein coupled receptors and integrins, suggesting that signalling is a key process in this life-cycle transition.

A total of 2,194 genes had detectable expression in at least one stage but not another and were therefore differentially expressed. We also used a pair-wise approach to analyse genes differentially expressed between the following life cycle stages: cercariae *vs.* 3-hour schistosomula, 3-hour schistosomula *vs* 24-hour schistosomula, and 24-hour schistosomula *vs.* adult. A total of 3,396 non-redundant transcripts (excluding alternative spliced forms) were differentially expressed (adjusted p-value<0.01) within the three pair wise comparisons (Table 4 and Table S3). An example showing differential expression between cercariae and 3-hour post-infection schistosomula is presented in Figure 6. To obtain a broad overview of the biological changes occurring at the gene expression level, we used Gene Ontology term enrichment to identify annotated functions and processes that were overrepresented in genes that were statistically (adjusted p-value<0.01) up- or down- regulated. Aerobic energy metabolism pathways were down regulated in schistosomules compared to cercariae and antioxidant enzymes were overrepresented in transcripts from adults. Three-hour post-infection schistosomula showed enrichment of transcripts involved in transcriptional regulation, G-protein coupled receptor (GPCR) and Wnt signalling pathways, cell adhesion and a considerable number of genes involved in potassium/sodium transport (Table S4). Most of the categories enriched at 3 hours post transformation persist through to 24 hours (e.g. GPCR signalling pathways). A total of 165 proteins are found to be associated with GPCR signalling pathways (annotated via GO). Of these, 30 and 18 were up regulated in 3 and 24 hours post-infection schistosomula, respectively, compared with cercariae.

**Table 4:** Number of differentially expressed genes.

Stage comparison	Up regulated	Down regulated	Total
Cercariae - 3 hour schistosomula	1,002	516	1,518
3 hour schistosomula - 24 hour schistosomula	433	595	1,028
24 hour schistosomula - adult	1,141	935	2,076

Figures refer to those genes with significant differential expression (adjusted p-value<0.01). NB the v5.0 assembly contains 10,852 genes.

**Figure 7:**
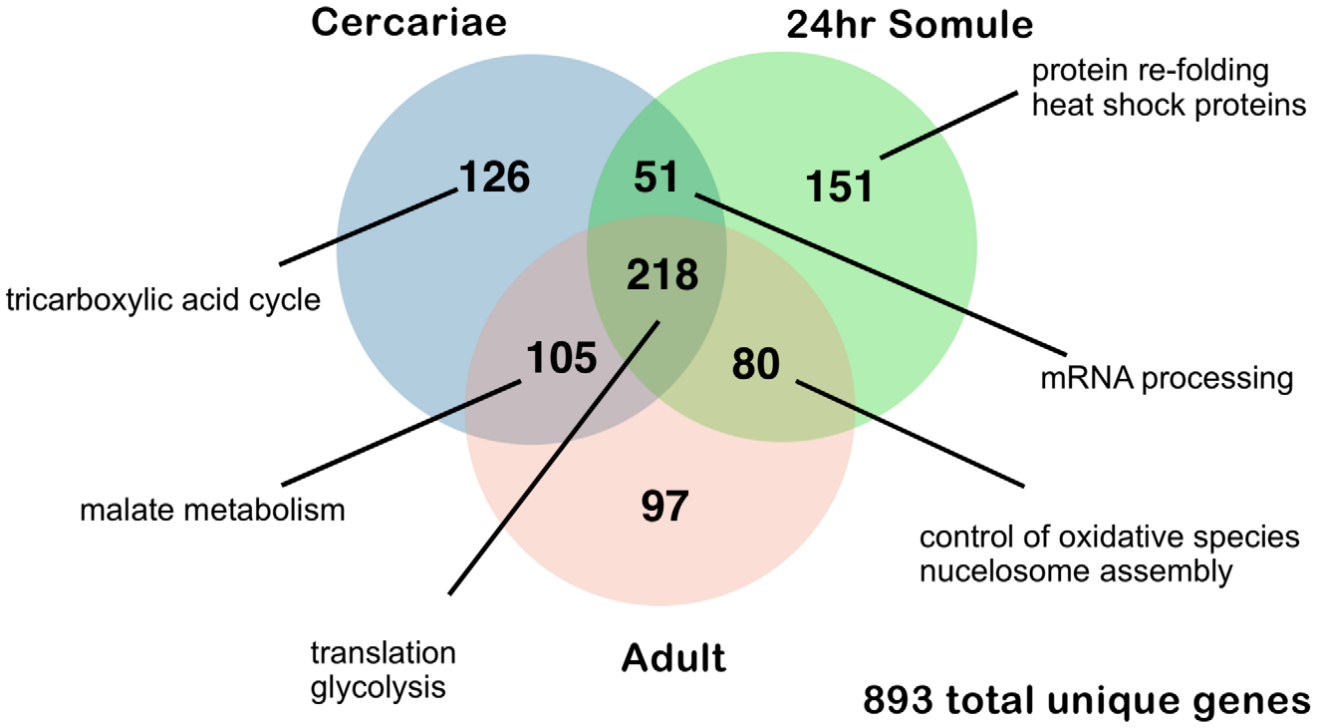
Genes with expression above the 95 percentile different in cercariae and intra-mammalian stages. Venn diagram represents the distribution of genes above 95 percentile of expression in 3 different life cycle stages of the parasite. Examples of the genes/processes found within these groups are discussed in the main text.

In order to investigate major processes occurring individually in each life cycle stage, we studied genes with expression above the 95 percentile in cercariae, 24-hour schistosomula and adults (Figure 7). Across the life cycle stages studied, some core cellular processes are consistently highly expressed, including glycolytic enzymes and protein translation but other broad changes are also apparent. Free-living cercariae utilise internal glycogen stores; accordingly genes involved in glycolysis and the tricarboxylic acid cycle (TCA) are highly expressed. After penetrating the skin and transforming into obligate endoparasites, the schistosomula switch to anaerobic metabolism [55], [56] before aerobic metabolism partly resumes in the adult. These events are also reflected in the transcriptome. At the schistosomulum stage there is a switch to high expression of L-lactate dehydrogenase, while TCA cycle transcription markedly decreases. As noted above, the cercariae and adult samples have relatively high contributions from the mitochondrial transcriptome (Figure S9) reflecting the high energy-demands of these two stages.

Other genes highly expressed in the schistosomula are involved in protein re-folding and chaperone function: 5 heat shock proteins (Smp_008545, Smp_035200, Smp_062420, Smp_072330, HSP70/Smp_106930) are among the top 50 most expressed genes at this stage and may reflect a response to the rapid temperature rise between fresh-water (∼28°C), in which the cercariae are found, and the warmer mammalian host (∼37°C). Within the host, schistosomes are exposed to potentially damaging reactive oxygen species produced during metabolism. Consistent with previous work [57] we found that antioxidant enzymes - particularly the peroxiredoxins (Prx1, Smp_059480 and Prx2, Smp_158110) - are highly expressed in adults, 24 hours after transformation and for Prx1, as early as 3 hours after transformation.

Our results highlight the advantages of RNA-seq transcriptome profiling, especially its ability to dramatically improve the gene annotation alongside accurately recording changes in gene expression.

## Discussion

In 2009 a draft genome of *S. mansoni* was published and provided a major resource for gene discovery and data mining. Our motivation for this study was to take *S. mansoni*'s genome to the next level, to systematically upgrade its draft sequence so that gene structures can be more accurately predicted and the genomic context of genes can be better explored. Although systematic manual finishing has occurred for some parasite genomes, it is not an economically viable option for most non-model organisms. The genome of *S. mansoni* is approximately 10 times larger that the genomes of protozoan parasites and is set in the context of a field that attracts less funding. Although additional “traditional” targeted, long-range capillary sequence was introduced, more than 40,000 gaps were closed simply by re-sequencing at deep coverage, from a low-polymorphic population of adult worms. Further substantial changes were made from re-evaluating existing genetic marker information. As a result, the genome is measurably more accurate and its continuity has been transformed; 81% of the data is now assembled into chromosomes.

We have also upgraded the annotation using deep coverage RNA-seq. Compared with the 2009 draft genome, the net change in the gene content is that there are now ∼900 fewer genes. However, 500 genes are new and more than 1600 low confidence or erroneous predictions have been removed. Across the genome, more than one third of genes now have new sequences. The value of the genome resource will therefore be tangibly improved: data mining approaches to identify genes will be more sensitive and trawling through kilobases of sequence for missing exons will be come less common.

Our results also highlight the major benefit of using RNA-seq for transcriptome profiling - its ability to dramatically improve the gene annotation, whilst accurately recording changes in gene expression. We see major expected changes, for example, the well described metabolic switch on host penetration, plus some previously overlooked ones, such as a battery of receptors up regulated at the onset of infection in the mammalian host. Our data also define with high resolution some of the important building blocks of the schistosome transcriptome – long transcripts, *cis* and *trans*-splicing, and for the first time, clear evidence of the *trans*-splicing being used to resolve polycistrons. By increasing the quality of the genome, we have increased the utility of our RNA-seq data and taken it well beyond the levels attainable by previous microarray approaches. Although only a broad view of gene expression changes are presented herein, the resolution of our analyses reflects the functional annotation that has been previously ascribed. The true value of these data will arise from their use within the context of genome databases such as GeneDB and SchistoDB to query the behaviour of specific genes or groups of genes.

The quality of a genome directly influences the uses to which it can be put and with many more, low-cost, draft-genome sequencing projects underway, the requirement for higher quality reference material, is increasing. Chain *et al.* 2009 recently defined several levels or standards for genome assemblies [58]. In the present study, we have taken an existing draft genome and demonstrated that in relatively modest period of time it can be upgraded to annotation-directed grade using second generation sequencing technology without the need for extensive manual finishing. The much improved genome assembly and gene structures, along with the expression data, are available at GeneDB and SchistoDB and will be an excellent resource not only for the helminth research community but also for in depth comparative genomics studies across metazoa.

## Supporting Information

Figure S1 **The frequency and length of newly inserted sequences at gaps.**
(PDF)

Figure S2 **The *S. mansoni*** **v5.0 genome assembly superimposed over a genetic linkage map** [**17**] **.** The numbers on the left of chromosomes are map distances in centimorgans, and the identifiers on the right of each chromosome denote contigs and scaffolds of assembly v5.0 (e.g. *6569_28* is contig 6569, which is assembled into scaffold 28). Lines connecting chromosomes indicate where an assembly scaffold contains contigs from two different chromosomes. There are multiple possible reasons for such occurrences, including repetitive sequences, assembly errors. All assembly ambiguities of this kind have been manually inspected and cannot be resolved using the current data.
(PDF)

Figure S3 **Analysis of male and female specific sequences.** Sequence data from both Z and W chromosomes assembled together but was resolved by aligning male (blue) and female (red) genome sequence reads. The arrowheads indicate Z-specific genetic linkage markers.
(PDF)

Figure S4 **Plot showing (A) transcript length and (B) number of exons for the three different categories of gene models transfered using the Rapid Annnotation Transfer Tool (RATT).** Outliers were not drawn in the boxplot.
(PDF)

Figure S5 **Codon usage of the (manually) curated genes and the 466 novel genes.**
(PDF)

Figure S6 **Correlation between replicate experiments.** Biological replicates are evaluated by calculating the Pearson's correlation for each pair of samples.
(PDF)

Figure S7 **Cumulative distribution of RNA-seq coverage (expressed as RPKM values, see *Methods*) for exons, introns, intergenic sequences and untranslated regions.**
(PDF)

Figure S8 **Correlation of RNA-seq data and microarray data.** The scatter plots show the coverage (Log_2_-transformed) of reads per probe location compared with normalized microarray intensities (Log_2_-transformed) from (A) Fitzpatrick *et al.* 2009 [54] and (B) Parker-Manuel *et al.* 2011 [53]. The graphs was generated using the smoothScatter function from the R software package [31].
(PDF)

Figure S9 **Relative gene expression levels for mitochondrial genes.** C = cercariae; 3S = 3 hour schistosomula; 24S = 24 hour schistosomula; A = adult.
(PDF)

Table S1 **Primers used for validation of trans-spliced (top) and polycistronic (bottom) transcripts.**
(XLS)

Table S2 **Putative polycistrons with a maximum intergenic distance of 200 bp and 2000 bp.**
(XLS)

Table S3 **Differentially expressed genes in the cercariae vs. 3 hr schistosomula comparison, 3 hr vs. 24 hr schistosomula comparison and 24 hr schistosomula vs. adult comparison.** Only significantly differentially expressed transcripts (adjusted p.value<0.01 – BH correction) are listed.
(XLS)

Table S4 **Gene Ontology (Biological Processes) enrichment for differentially expressed genes in the cercariae vs. 3 hr schistosomula comparison, 3 hr vs. 24 hr schistosomula comparison and 24 hr schistosomula vs. adult comparison.** The top 20 hits are shown.
(XLS)

Text S1 **Supplementary Materials and Methods.**
(DOC)
