# Supplementary figures and images for: A Systematically Improved High Quality Genome and Transcriptome of the Human Blood Fluke Schistosoma mansoni
Source: PLoS Negl Trop Dis. 2012 Jan 10;6(1):e1455. doi: 10.1371/journal.pntd.0001455 (PMC3254664; doi:10.1371/journal.pntd.0001455)

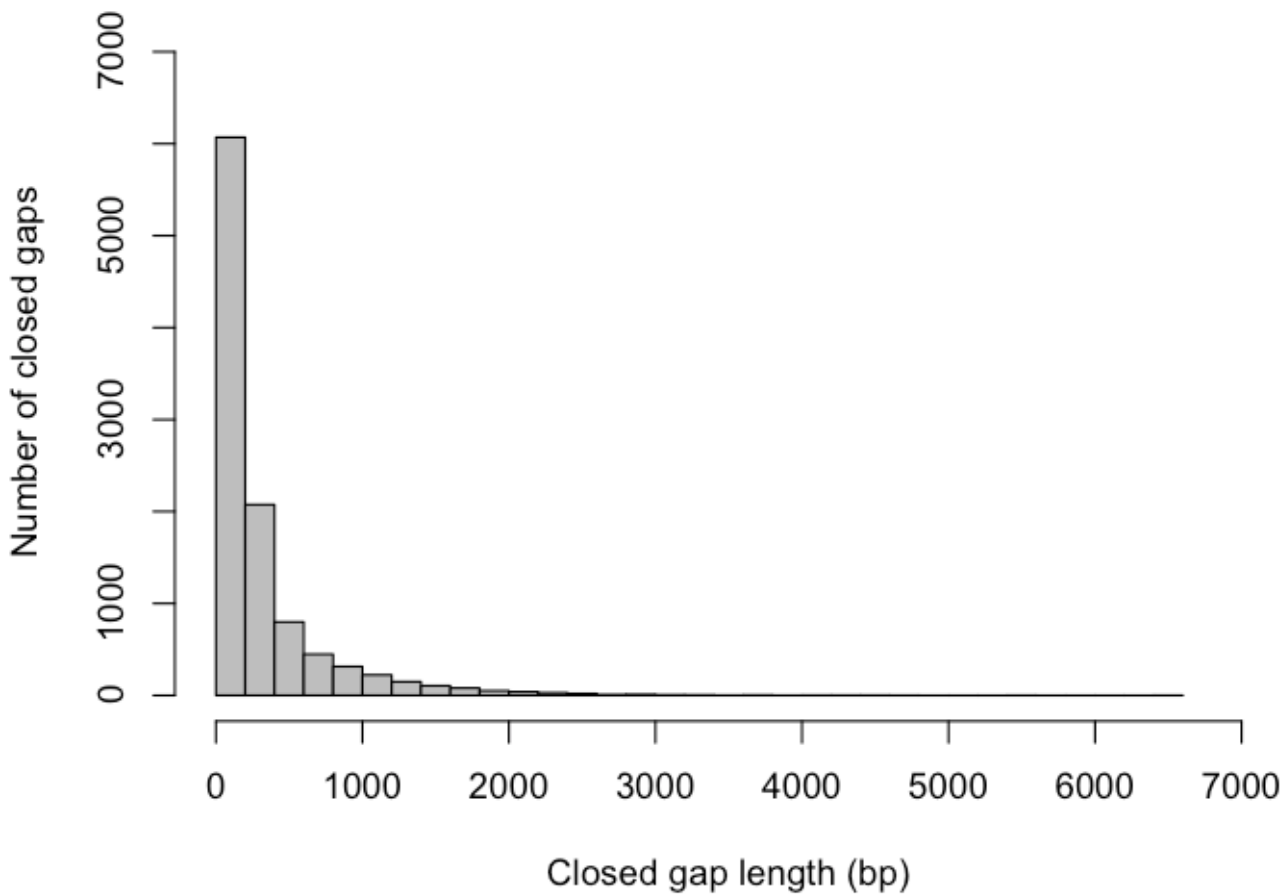

Supplement: Figure S1 — The frequency and length of newly inserted sequences at gaps. (PDF) [file pntd.0001455.s001.pdf]

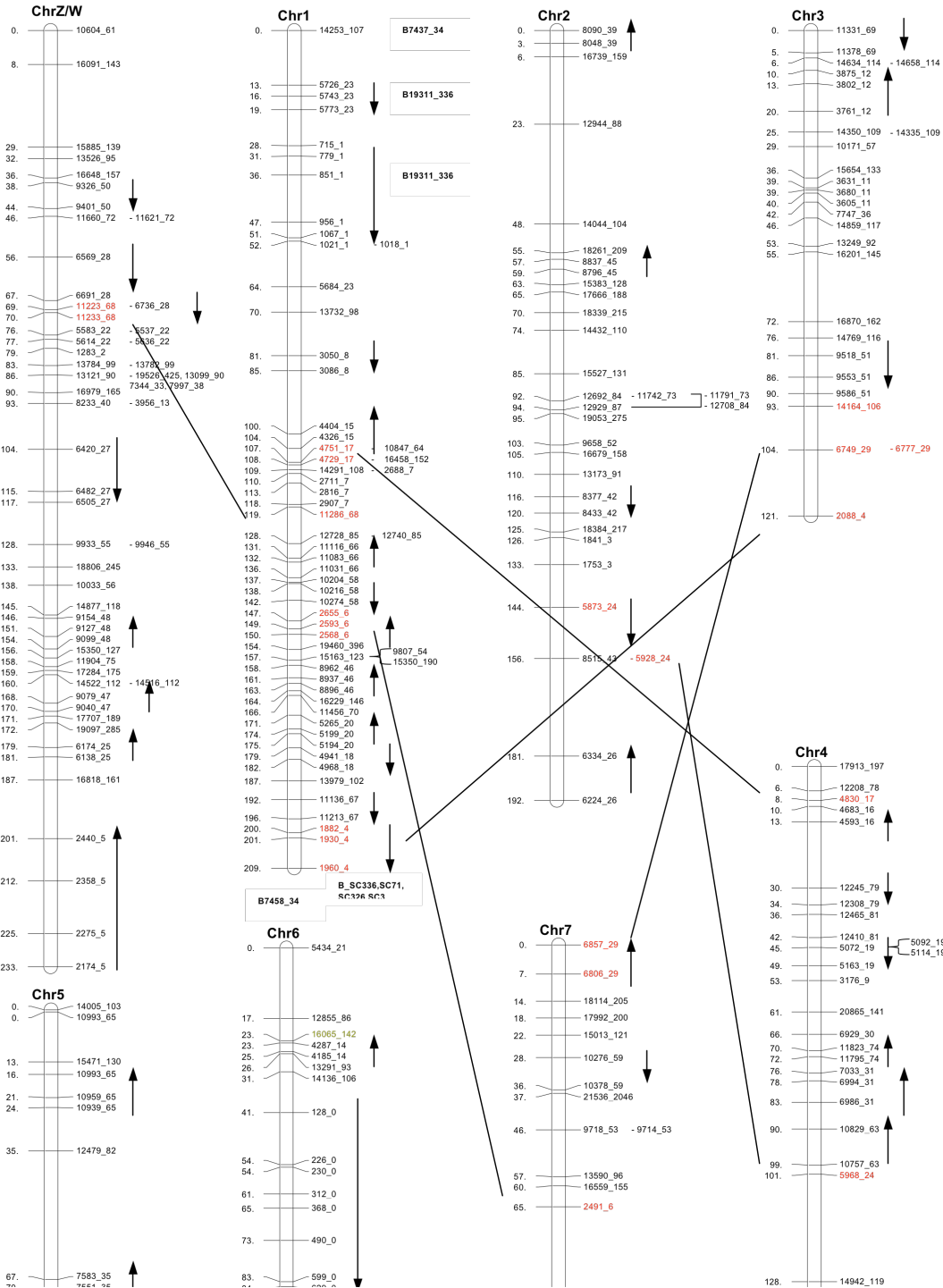

Supplement: Figure S2 — The S. mansoni v5.0 genome assembly superimposed over a genetic linkage map [17] . The numbers on the left of chromosomes are map distances in centimorgans, and the identifiers on the right of each chromosome denote contigs and scaffolds of assembly v5.0 (e.g. 6569_28 is contig 6569, which is assembled into scaffold 28). Lines connecting chromosomes indicate where an assembly scaffold contains contigs from two different chromosomes. There are multiple possible reasons for such occurrences, including repetitive sequences, assembly errors. All assembly ambiguities of this kind have been manually inspected and cannot be resolved using the current data. (PDF) [file pntd.0001455.s002.pdf]

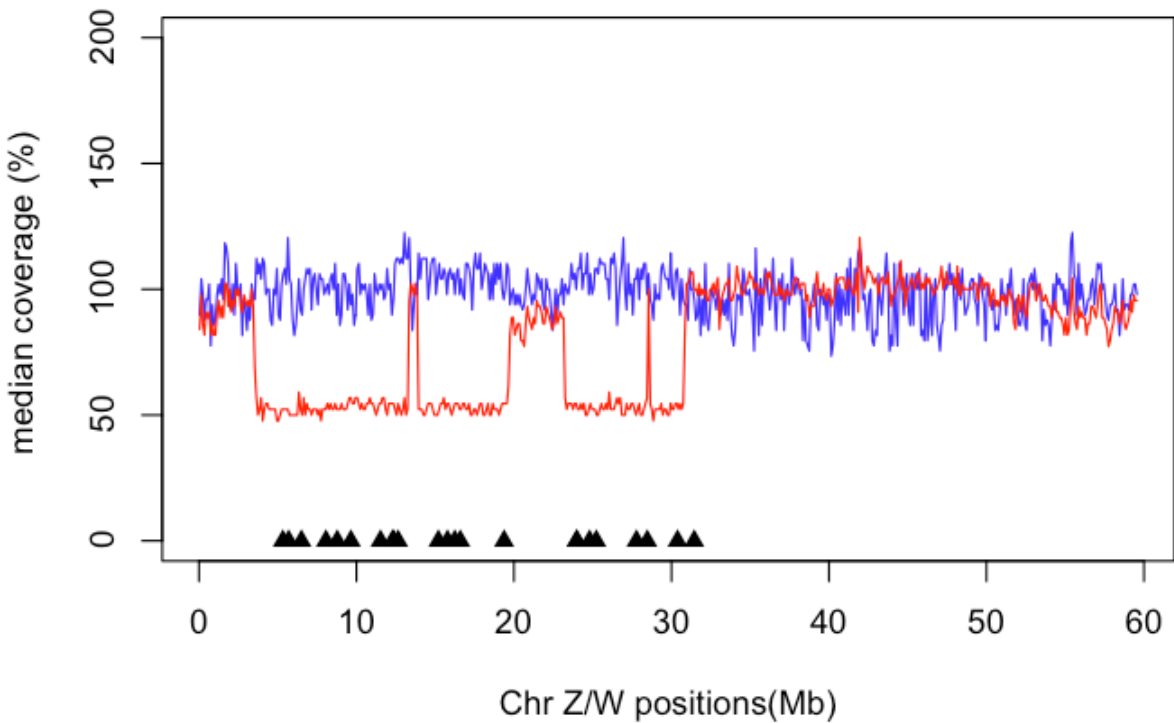

Supplement: Figure S3 — Analysis of male and female specific sequences. Sequence data from both Z and W chromosomes assembled together but was resolved by aligning male (blue) and female (red) genome sequence reads. The arrowheads indicate Z-specific genetic linkage markers. (PDF) [file pntd.0001455.s003.pdf]

**A**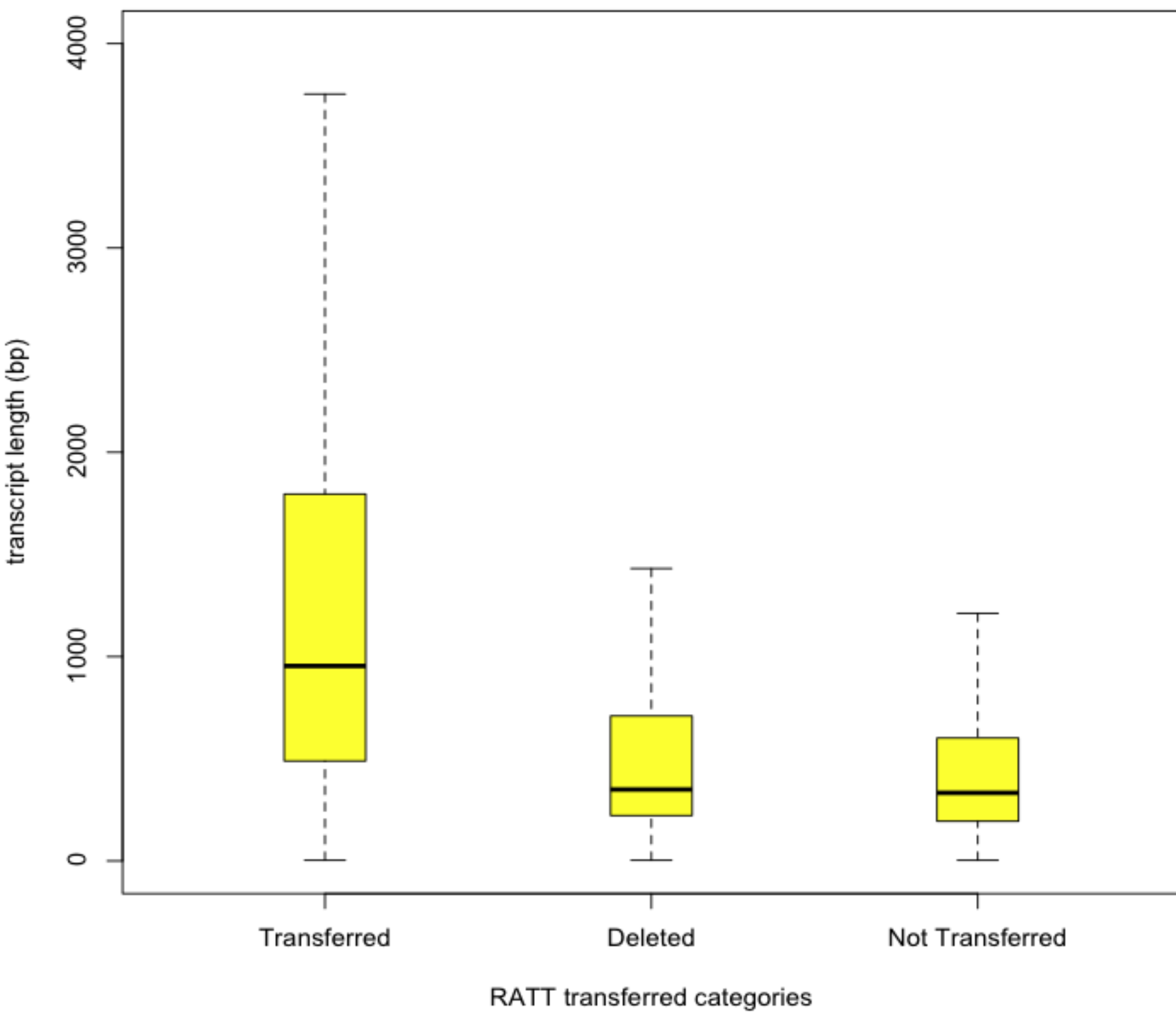**B**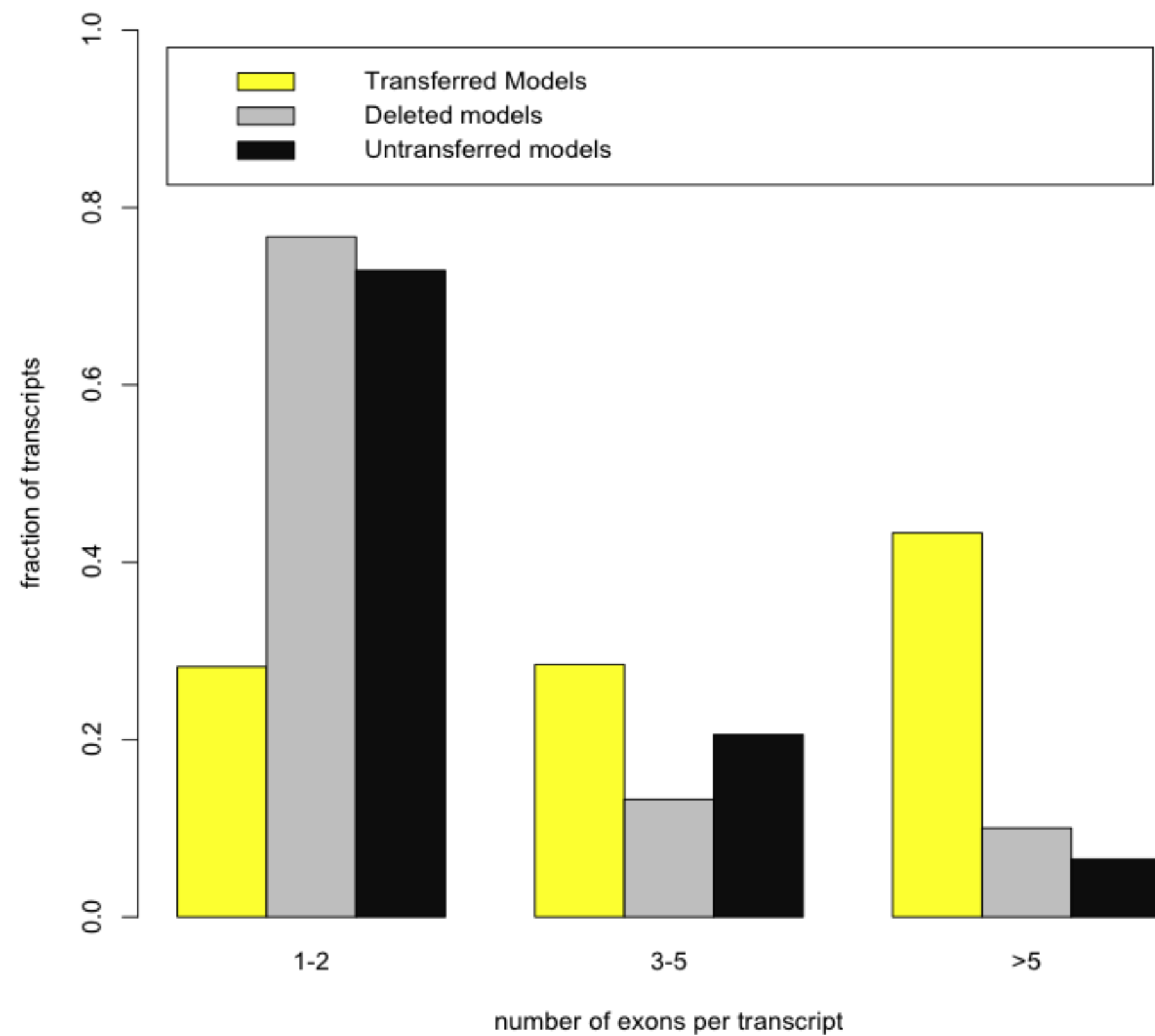

Supplement: Figure S4 — Plot showing (A) transcript length and (B) number of exons for the three different categories of gene models transfered using the Rapid Annnotation Transfer Tool (RATT). Outliers were not drawn in the boxplot. (PDF) [file pntd.0001455.s004.pdf]

Frequency of optimal codons(%)

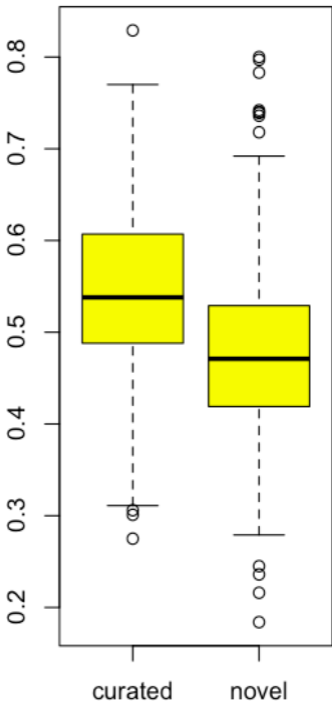

Supplement: Figure S5 — Codon usage of the (manually) curated genes and the 466 novel genes. (PDF) [file pntd.0001455.s005.pdf]

**CERCARIAE**

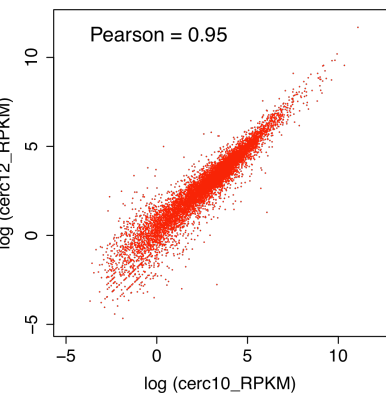

**CERCARIAE**

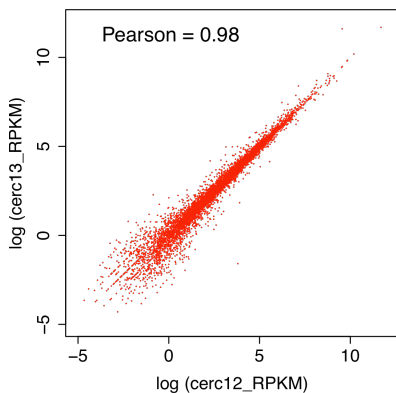

**CERCARIAE**

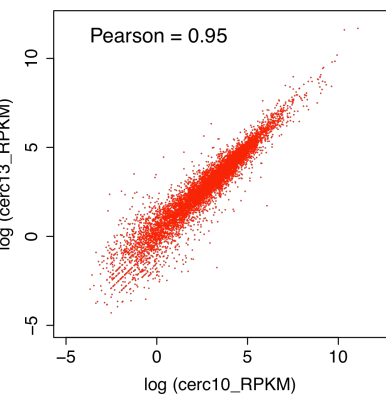

**3hr SOMULES(mech)**

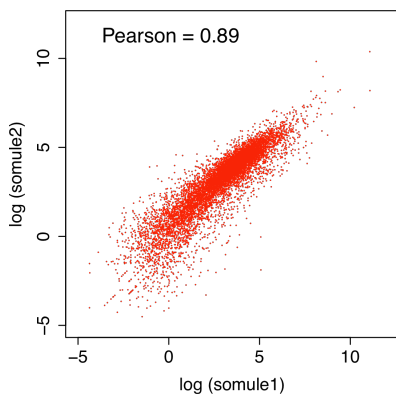

**24hr SOMULES(mech)**

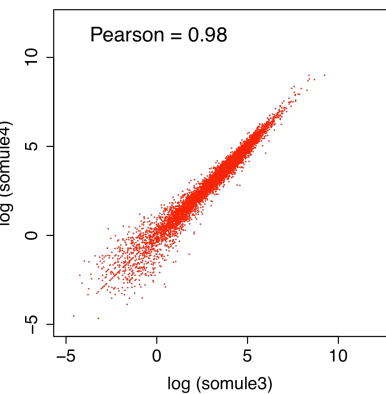

Supplement: Figure S6 — Correlation between replicate experiments. Biological replicates are evaluated by calculating the Pearson's correlation for each pair of samples. (PDF) [file pntd.0001455.s006.pdf]

**Estimation of the minimum RPKM value  
to discriminate between signal and noise**

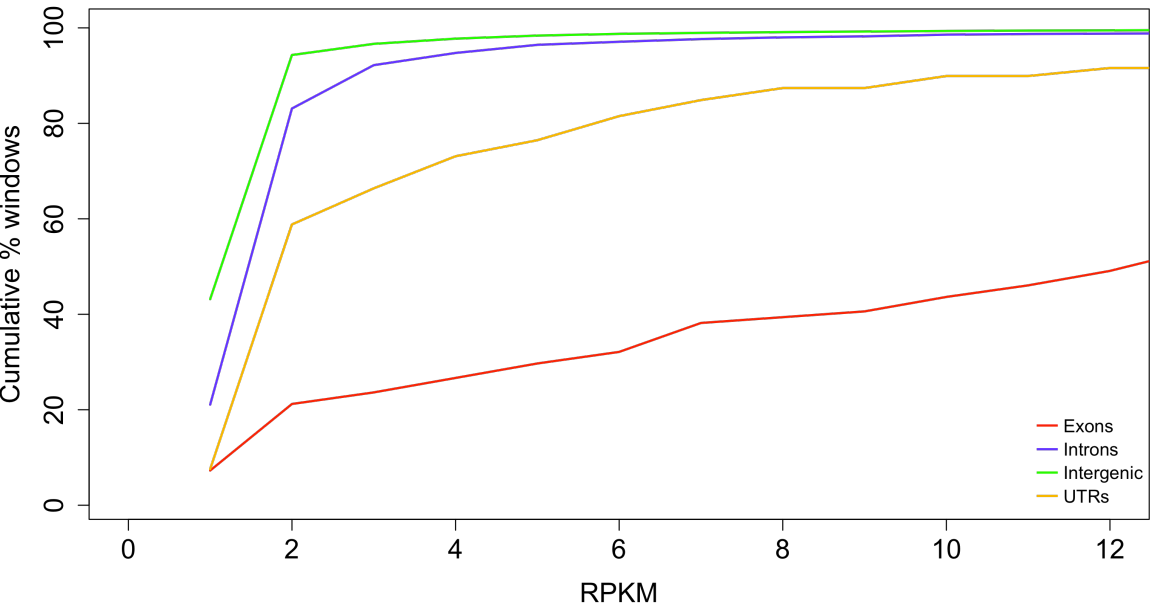

Supplement: Figure S7 — Cumulative distribution of RNA-seq coverage (expressed as RPKM values, see Methods) for exons, introns, intergenic sequences and untranslated regions. (PDF) [file pntd.0001455.s007.pdf]

**A**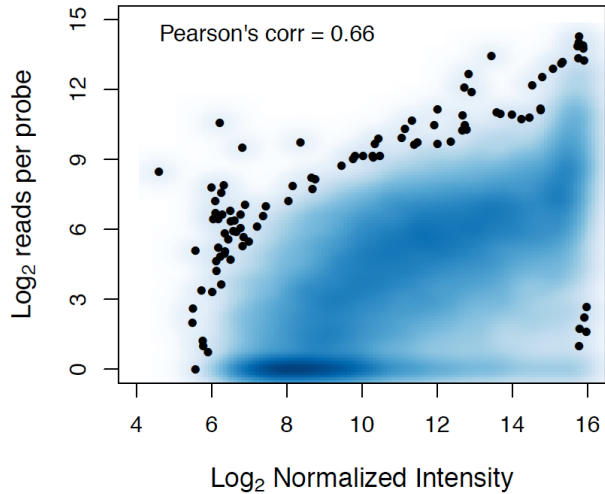**B**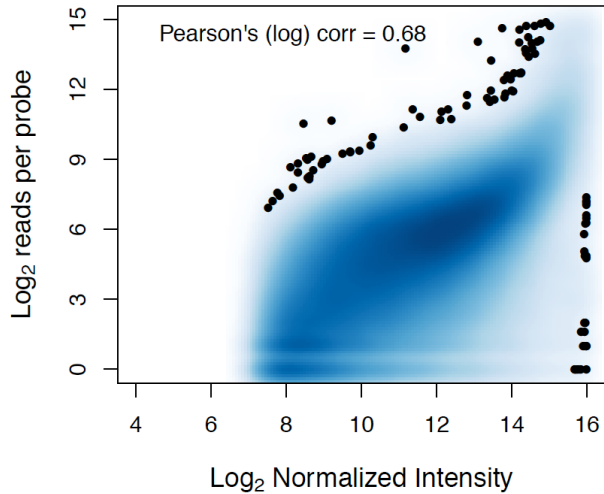

Supplement: Figure S8 — Correlation of RNA-seq data and microarray data. The scatter plots show the coverage (Log2-transformed) of reads per probe location compared with normalized microarray intensities (Log2-transformed) from (A) Fitzpatrick et al. 2009 [54] and (B) Parker-Manuel et al. 2011 [53]. The graphs was generated using the smoothScatter function from the R software package [31]. (PDF) [file pntd.0001455.s008.pdf]

Relative normalized reads

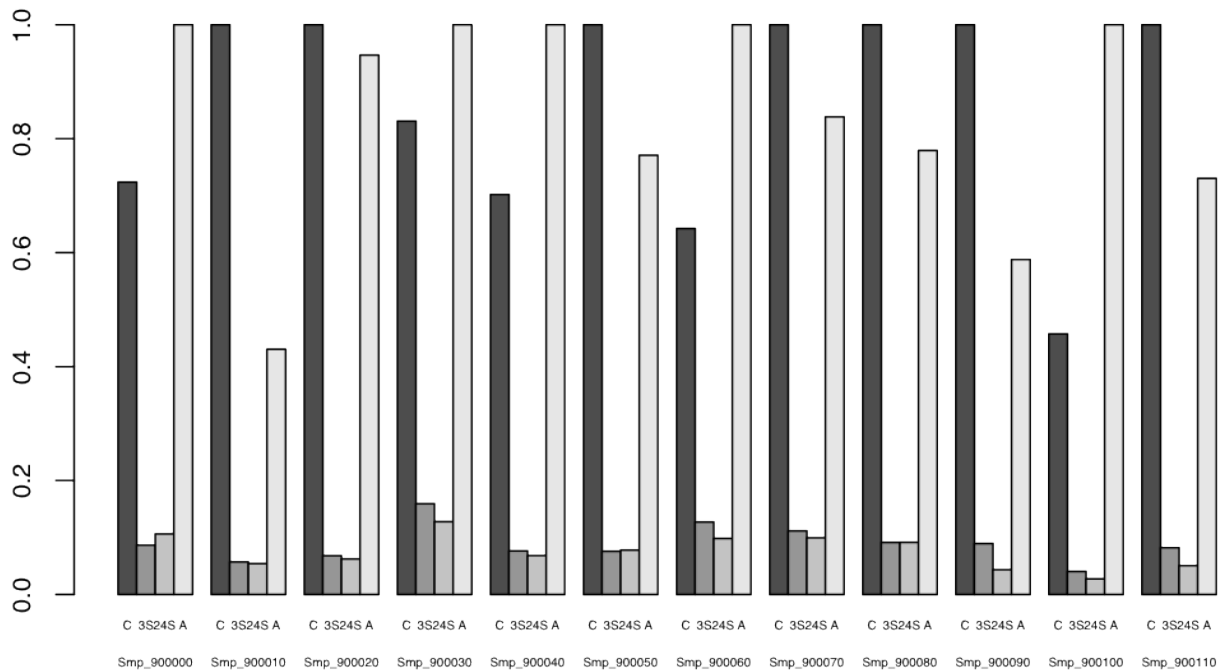

Supplement: Figure S9 — Relative gene expression levels for mitochondrial genes. C = cercariae; 3S = 3 hour schistosomula; 24S = 24 hour schistosomula; A = adult. (PDF) [file pntd.0001455.s009.pdf]
