# Supplementary material for: A Systematically Improved High Quality Genome and Transcriptome of the Human Blood Fluke Schistosoma mansoni
Source: PLoS Negl Trop Dis. 2012 Jan 10;6(1):e1455. doi: 10.1371/journal.pntd.0001455 (PMC3254664; doi:10.1371/journal.pntd.0001455)
Supplement: Text S1 — Supplementary Materials and Methods. (DOC) [file pntd.0001455.s014.doc]

# Supplementary Materials and Methods

## Parasite material, library preparation and sequencing.

### Isolation of low-polymorphism DNA from male adult worms

*Biomphalaria glabrata* snails were individually exposed to single miracidia of *S. mansoni* NMRI (Puerto Rican origin) in 24-well culture plates for 30 min. After group housing for five weeks in small plastic aquaria at 27°C, each snail was tested individually for cercarial shedding in 24-well culture plates under a strong light. Those releasing cercariae (~30%) were housed subsequently in single beakers of pond water as numbered “clones” (although they could still exhibit allelic variation at each gene locus); no attempt was made to determine sex at this stage. The cercariae from each single miracidium infection were then used to infect groups of NMRI-strain mice (Harlan Laboratories, Bicester, UK) percutaneously via the shaved abdomen, using 250 cercariae per animal, on a number of occasions to bulk-up the biomass. After five weeks the adult worms were perfused from the portal system of each mouse using RPMI medium (Gibco Invitrogen, UK), let to sediment by gravity and washed several times with RPMI medium to remove erythrocytes. Any mouse hair or tissue fragments were removed using tweezers under a dissecting microscope, at which time worm sex was determined by morphology (~50:50, male and female) before the male or female worms of one “clone” were combined in a minimal volume of medium and frozen in liquid N_2_ and kept at -80°C until used. In this way samples of several hundred genetically homogeneous worms were obtained for DNA extraction [[1](#_ENREF_1)].

### DNA extraction and library preparation for sequencing

Frozen adult worms were ground in liquid N_2_ using a pestle and mortar then resuspended in QIAgen Cell Resuspension Buffer (QIAgen, UK). DNA extraction was performed using a DNeasy Blood and Tissue kit (QIAgen, UK) following manufacturer instructions. Quality control of DNA was assessed using a NanoDrop ND-1000 UV-Vis spectrophotometer and quantified by gel electrophoresis relative to markers of known quantity. Libraries for DNA sequencing were prepared as previously described [[2](#_ENREF_2)]. Male libraries were sequenced in eight lanes while females were sequenced in one lane. Both sequencing runs produced 108 base paired-reads using an Illumina Genome Analyzer IIx. Sequence data produced from both male and female worms were submitted to ENA under accession number ERP000385.

### Parasite RNA

*S. mansoni* (NMRI strain, Puerto Rican origin) eggs were kindly supplied by Prof. Michael J. Doenhoff (University of Nottingham, Nottingham, U.K.). Miracidia were allowed to hatch in non-chlorinated water and were separated phototropically. *B. glabrata* snails were infected with 2-6 mixed sex miracidia each and kept in the dark for five weeks prior to shedding cercariae phototropically. Mechanically transformed schistosomula were obtained using a modified version of Brink *et al.* [[3](#_ENREF_3)]. Briefly, freshly shed cercariae, while in non-chlorinated aquarium water, were cooled on ice for 30 minutes, centrifuged at 1000 *g* for 10 minutes at 4°C and then resuspended in 10 ml of high glucose Dulbecco’s Modified Eagle’s medium (DMEM) (Sigma, U.K.), 100 ng/ml penicillin–streptomycin (Sigma, U.K.) and complemented with L-glutamine (Sigma, U.K.). Cercariae were then vigorously shaken for approximately 30 seconds in a vortex mixer and subjected to 13-15 passages through a 21G syringe needle. Separation of heads and tails was performed using a 65% Percoll gradient (Sigma, UK) in DMEM. Schistosomula preparations were incubated at 37°C and 5% CO_2_ for 3 hours or 24 hours in high glucose DMEM, 100 ng/ml penicillin–streptomycin, L-glutamine, 10% fetal calf serum (FCS) (PAA, UK) and 1% Hepes buffer (PAA, UK). Adult worms (perfused at 7-weeks after infection) were obtained from infected hamsters and placed in RNALater (Ambion, UK) at -80°C until RNA extraction.

Total RNA from parasite material was isolated using TRIzol (Invitrogen, UK) according to manufacturer specifications with the exception of cercariae samples, where a modified TRIzol (Invitrogen) / RNeasy (Qiagen, UK) protocol [[4](#_ENREF_4)] was used instead. After extraction, RNA quality was assessed using an Agilent RNA 6000 Nano - Bioanalyzer and quantified using a NanoDrop ND-1000 UV-Vis spectrophotometer.

RNA-seq libraries were prepared using the protocol described in [[5](#_ENREF_5)] with the following modifications: i) mRNA was fragmented by incubating samples in 5x Fragmentation Buffer (Ambion, UK) at 70°C for 5 minutes followed by removal of fragmentation buffer using sodium acetate/isopropanol precipitation of RNA; ii) after second strand cDNA synthesis, the Illumina Genomic DNA Sample Preparation Kit (Illumina, UK) was used according to manufacturer instructions and with modifications previously described in [[6](#_ENREF_6)]. These libraries were sequenced as 76-base read-pairs using the Illumina Genome Analyzer IIx platform. A total of ten flow-cell lanes were generated: cercariae (3 lanes), adult (1 lane), schistosomula (6 lanes). Adaptor contamination was removed from the reads and rRNA contamination was in all cases < 1% (data not shown). All sequenced samples were submitted to ArrayExpress (http://www.ebi.ac.uk/arrayexpress/) under the accession number E-MTAB-451.

## Genome assembly and manual improvement.

The Arachne assembler (version 3.2, [[7](#_ENREF_7)]) was used to produce a new assembly using the existing capillary reads from the published draft (v4.0) genome assembly [[8](#_ENREF_8)], supplemented with an additional ~90,000 fosmid and BAC end sequences. FISH-mapped BACs [[8](#_ENREF_8)] were also end-sequenced and 438 passed reads were incorporated into the assembly. Illumina reads were assembled using IMAGE [[9](#_ENREF_9)], which was run on the assembly (excluding singlet contigs less than 20 kb) with 33 iterations of different *k*-mer sizes with overhang parameter of 100 and extension parameter of 10. The sequence information of 243 published linkage markers [[10](#_ENREF_10)] of *S. mansoni* were retrieved and used as anchors within the assembly by incorporating them as *faux* capillary reads. Scaffolds containing these reads were ordered, orientated and merged (with 2 kb gaps in between) into chromosomes.

Contigs within scaffolds were joined together automatically with 200 N’s, based on paired-read information. The order and orientation of contigs and scaffolds was verified using capillary read pairs, the position of FISH-mapped BACs [[8](#_ENREF_8)] and linkage groups [[10](#_ENREF_10)]. Where identified, misassembled sequences were reassembled correctly. In total, 2,062 automatically closed joins were manually verified and corrected where required. Contigs containing known telomeric repeat sequences (TTAGGG) [[11](#_ENREF_11)] were extended by oligo-walking pUC clones until unique sequence was identified. Where the unique sequence was linked to a known marker, the telomere could be placed onto a chromosome.

The proportion of the combined Z/W chromosomal sequence that was specific to either sex chromosome within the new assembly was determined by analyzing the difference in coverage in mapped sequence data from male (containing two Z chromosomes) and female-specific libraries (containing both Z and W chromosomes). Reads were aligned using SSAHA2 [[12](#_ENREF_12)] and depth of coverage analyzed in non-overlapping 100 kb windows. Chromosome 1 was also analyzed to establish the median baseline depth of read coverage (45x and 44x for male and female data, respectively). Z-specific regions should have a depth of coverage equivalent to approximately half that of autosomes in a female sample, but approximately equivalent to autosomes in the male samples. To determine putative Z-specific regions in the rest of the assembly, we searched for windows of 100 kb that had at least 36x depth of coverage in the male (i.e. >= 0.8x autosomal coverage) but less than 24x depth of coverage in the female sample (i.e. < 0.6x autosomal coverage). W-specific regions should have a depth of coverage equivalent to approximately half that of autosomes in a female sample, but be completely absent in male samples. To determine putative W -specific regions within the assembly, we searched for windows of 100 kb that had at least 20x depth of coverage in the female (i.e. >= 0.45x autosomal coverage) but less than 10x depth coverage in the male sample (i.e. < 0.22 x autosomal coverage).

Manual finishing is ongoing. However, prior to finishing a snapshot of the data was taken. Except where indicated, all subsequent analyses reported in the present study, refer to the stable snapshot dataset (*S. mansoni* genome v5.0).

## Transferring protein coding gene annotation

To transfer the existing annotation to the latest reference sequence we used RATT [[13](#_ENREF_13)]; this defines apparent synteny between both assemblies and transfers the annotation features, such as gene models, to the new assembly. Due to its size, the old genome assembly (v4.0) was split into four parts and then rejoined after mapping. Synteny blocks were identified using the repetitive option (-q and –r) of the software and a word size of 30. In a further checking step, underlying problems that are uncovered by RATT were corrected in gene models (wrong start/stop codons and splice sites).

## Gene finding using RNA-seq

Each lane of RNA-seq reads was independently aligned to the genome using TopHat (version 1.1.0; with default parameters except -a 4 -r 32 --min-intron 25 --max-intron 35000) [[14](#_ENREF_14)] and the resulting binary sequence alignment mapping (BAM) files used as the input for the gene finder Cufflinks (combining results of version 0.8.2 and version 0.9.1; parameters --inner-dist-mean 50 -Q 30 were used) [[15](#_ENREF_15)]. Transcript fragments with less than 10x average read depth coverage were excluded from subsequent analyses. During manual inspection many single exon gene models appeared to be false positives. Hence transcript fragments containing open reading frames of less than 50 codons were removed from the predicted set. Such short genes are extremely rare in this organism making up less than 0.1% of the models on version 4.0 of the genome.

Jigsaw (version 3.2.10; with parameters –l) [[16](#_ENREF_16)] was used to combine existing models and transcript fragments from Cufflinks. Where the conceptual translation of the combined model was longer than the previous models the combined models automatically replaced those within the annotation database, GeneDB (http://www.genedb.org). As a consequence of using this approach, the small introns at the start of some transcripts were preserved while the frameshifts were corrected. The remainder of the transcript fragments that could not be built into gene models has been loaded into GeneDB (http://www.genedb.org/Homepage/Smansoni) as browsable evidence tracks for further manual curation. Novel complete transcripts with more than 50 codons were annotated using BLASTx against a non-redundant database (NCBI nr) and InterPro [[17](#_ENREF_17)].

## Identification and validation of trans-spliced genes and polycistrons

RNA-seq data was screened for reads containing the 36-nucleotide sequence corresponding to *S. mansoni* splice-leader (SL) [[18](#_ENREF_18)]. Subsequently, the SL sequence was clipped off the reads and the remainder of the read (and its mate pair) was mapped to the genome using SSAHA2 [[12](#_ENREF_12)] allowing putative *trans*-spliced acceptor sites to be identified. *Trans*-splicing acceptor sites can fall in four different places: upstream of start of gene, in an exon, in an intron or downstream of a gene. In order to identify polycistronic units, we looked for genes found within 200 bp and up to 2000 bp upstream of the putative *trans*-spliced site. Only those genes with a *trans*-splicing acceptor site (indicated by the SL-reads) upstream (maximum of 500 bases) of the start of the gene, or within the gene boundaries (either intron or exon), were considered as putative *trans*-spliced transcripts. For the identification of polycistronic transcripts, we looked for genes found within 200 bp, and up to 2000 bp upstream, of the putative *trans*-spliced gene. Where a gene was found within the specified distance, the gene pair was catalogued as a putative polycistron. In order to validate both the *trans*-splicing and polycistronic predictions, 1 ug of DNase-treated (Ambion, UK) total RNA from 24-hour schistosomula was used for a reverse transcription reaction using Superscript II (Invitrogen, UK) according to manufacturers specifications. Single-strand cDNA was then used for PCR using Fast Cycling PCR kit (Qiagen, UK). For the *trans*-splicing validation, PCR amplifications were performed with a forward primer SL1 and gene specific reverse primers (Table S1). The already known *trans*-spliced phosphopyruvate hydratase gene (Smp_024110) reported in [[19](#_ENREF_19)] was used as a positive control and Smp_045200 as a negative control. For validation of polycistrons, each polycistron was subjected to two PCR reactions; the first one evaluates the presence of a transcript containing the intergenic region (using gene specific primers from both upstream and downstream genes) while the second evaluates the presence of the *trans*-spliced gene (using the SL1 and a gene specific primers - Table S1). We used the previously suggested polycistron Ubiquinol-cytochrome-c-reductase (UbCRBP) and phosphopyruvate hydratase (Smp_024120 and Smp_024110 respectively) reported in [[19](#_ENREF_19)] as a positive control. In both cases, PCR products were analyzed in a 2% agarose gel and the Hyperladder IV (Bioline Ltd, UK) was used as a DNA size reference. The agarose gel was stained in an ethidium bromide solution and bands were visualized under UV light. The polycistron PCR products were verified by Sanger capillary sequencing.

## Quantification of RNA-seq and correlation with microarray data

For gene expression quantification, RNA-seq reads were aligned to the reference genome using SSAHA2 [[12](#_ENREF_12)] (version 2.5.1 with default parameters except -solexa -pair 1,30000) and filtered with a minimum mapping score of 10. Reads per exon were calculated using BEDTools [[20](#_ENREF_20)] and then summarized to provide total number of reads per gene. From these, RPKM (reads per Kilobase per million mapped reads [[5](#_ENREF_5)]) values could be calculated. In order to differentiate signal from background noise, we calculated an RPKM value for non-overlapping 500 bp windows (for example, from position 1 to position 500 is one segment, from position 501 to position 1000 is another segment and so on) across exons, introns, untranslated regions (UTRs – considered to be 100 bp up- and downstream of a given annotated gene) and intergenic regions of the Schisto_mansoni.Chr_1.unplaced.SC_0010 scaffold and compared their RPKM values. Figure S6 shows the cumulative distribution of RPKM values for each sequences type. If each of these windows are considered as a potential expressed unit, by choosing an RPKM cut-off value of 2, 90-95% of the introns and intergenic regions are removed compared to only ~23% of exonic regions. Since these exonic regions have the same cumulative RPKM as non-coding regions they can be considered as non-expressed.

We used published microarray data [[21](#_ENREF_21)] for the cercariae stage to validate our RNA-seq results. In order to produce a correlation between oligonucleotide probes and gene models, the 389,211 60-*mer* probes [[21](#_ENREF_21)] or the 35,078 unique 50-mer probes [[22](#_ENREF_22)], from each array were mapped to the genome using SSAHA2 [[12](#_ENREF_12)] and only perfect matches (100% identity) that unambiguously matched one location in the genome were selected for subsequent analysis. Probe’s coordinates where used to find the number of RNA-seq reads covering each probe location. Log2 values of both normalized microarray intensities and RNA-seq reads were used to calculate the Pearson’s correlation values (0.66 and 0.68) and a scatter plot representing this correlation is presented in Figure S7.

## Differential expression and Gene Ontology term enrichment analysis

Differential expression analysis was performed with the EdgeR package [[23](#_ENREF_23)] implemented in Bioconductor [[24](#_ENREF_24)] using uniquely mapped reads per gene as input. Only transcripts that were found to be differentially expressed with an adjusted p-value <0.01 (adjusted using a method for multiple testing [[25](#_ENREF_25)]) were considered significant and used for downstream analysis.

Gene Ontology (GO) term enrichment analysis was performed with TopGO [[26](#_ENREF_26)] implemented in Bioconductor [[24](#_ENREF_24)] using statistically significant (adjusted p-value <0.01) differentially expressed genes as input. Up- and downregulated genes were considered independently; only the top 20 (provided p-value <0.05) most significant enriched terms were considered for analysis.

**REFERENCES**

1. Gasser RB, Morahan G, Mitchell GF (1991) Sexing single larval stages of *Schistosoma mansoni* by polymerase chain reaction. Mol Biochem Parasitol 47: 255-258.

2. Kozarewa I, Ning Z, Quail MA, Sanders MJ, Berriman M*, et al.* (2009) Amplification-free Illumina sequencing-library preparation facilitates improved mapping and assembly of (G+C)-biased genomes. Nat Methods 6: 291-295.

3. Brink LH, McLaren DJ, Smithers SR (1977) *Schistosoma mansoni*: a comparative study of artificially transformed schistosomula and schistosomula recovered after cercarial penetration of isolated skin. Parasitology 74: 73-86.

4. Hoffmann KF, Johnston DA, Dunne DW (2002) Identification of *Schistosoma mansoni* gender-associated gene transcripts by cDNA microarray profiling. Genome Biol 3: RESEARCH0041.

5. Mortazavi A, Williams BA, McCue K, Schaeffer L, Wold B (2008) Mapping and quantifying mammalian transcriptomes by RNA-Seq. Nat Methods 5: 621-628.

6. Quail MA, Kozarewa I, Smith F, Scally A, Stephens PJ*, et al.* (2008) A large genome center's improvements to the Illumina sequencing system. Nat Methods 5: 1005-1010.

7. Batzoglou S, Jaffe DB, Stanley K, Butler J, Gnerre S*, et al.* (2002) ARACHNE: a whole-genome shotgun assembler. Genome Res 12: 177-189.

8. Berriman M, Haas BJ, LoVerde PT, Wilson RA, Dillon GP*, et al.* (2009) The genome of the blood fluke *Schistosoma mansoni*. Nature 460: 352-358.

9. Tsai IJ, Otto TD, Berriman M (2010) Improving draft assemblies by iterative mapping and assembly of short reads to eliminate gaps. Genome Biol 11: R41.

10. Criscione CD, Valentim CL, Hirai H, LoVerde PT, Anderson TJ (2009) Genomic linkage map of the human blood fluke *Schistosoma mansoni*. Genome Biol 10: R71.

11. Hirai H, LoVerde PT (1996) Identification of the telomeres on *Schistosoma mansoni* chromosomes by FISH. J Parasitol 82: 511-512.

12. Ning Z, Cox AJ, Mullikin JC (2001) SSAHA: a fast search method for large DNA databases. Genome Res 11: 1725-1729.

13. Otto TD, Dillon GP, Degrave WS, Berriman M (2011) RATT: Rapid Annotation Transfer Tool. Nucleic Acids Res 39: e57.

14. Trapnell C, Pachter L, Salzberg SL (2009) TopHat: discovering splice junctions with RNA-Seq. Bioinformatics 25: 1105-1111.

15. Trapnell C, Williams BA, Pertea G, Mortazavi A, Kwan G*, et al.* (2010) Transcript assembly and quantification by RNA-Seq reveals unannotated transcripts and isoform switching during cell differentiation. Nat Biotechnol 28: 511-515.

16. Allen JE, Majoros WH, Pertea M, Salzberg SL (2006) JIGSAW, GeneZilla, and GlimmerHMM: puzzling out the features of human genes in the ENCODE regions. Genome Biol 7 Suppl 1: S9 1-13.

17. Hunter S, Apweiler R, Attwood TK, Bairoch A, Bateman A*, et al.* (2009) InterPro: the integrative protein signature database. Nucleic Acids Res 37: D211-215.

18. Rajkovic A, Davis RE, Simonsen JN, Rottman FM (1990) A spliced leader is present on a subset of mRNAs from the human parasite *Schistosoma mansoni*. Proc Natl Acad Sci U S A 87: 8879-8883.

19. Davis RE, Hodgson S (1997) Gene linkage and steady state RNAs suggest trans-splicing may be associated with a polycistronic transcript in *Schistosoma mansoni*. Mol Biochem Parasitol 89: 25-39.

20. Quinlan AR, Hall IM (2010) BEDTools: a flexible suite of utilities for comparing genomic features. Bioinformatics 26: 841-842.

21. Parker-Manuel SJ, Ivens AC, Dillon GP, Wilson RA (2011) Gene Expression Patterns in Larval *Schistosoma mansoni* Associated with Infection of the Mammalian Host. PLoS Negl Trop Dis 5: e1274.

22. Fitzpatrick JM, Peak E, Perally S, Chalmers IW, Barrett J*, et al.* (2009) Anti-schistosomal intervention targets identified by lifecycle transcriptomic analyses. PLoS Negl Trop Dis 3: e543.

23. Robinson MD, Oshlack A (2010) A scaling normalization method for differential expression analysis of RNA-seq data. Genome Biol 11: R25.

24. Gentleman RC, Carey VJ, Bates DM, Bolstad B, Dettling M*, et al.* (2004) Bioconductor: open software development for computational biology and bioinformatics. Genome Biol 5: R80.

25. Benjamini Y, Drai D, Elmer G, Kafkafi N, Golani I (2001) Controlling the false discovery rate in behavior genetics research. Behav Brain Res 125: 279-284.

26. Alexa A, Rahnenfuhrer J, Lengauer T (2006) Improved scoring of functional groups from gene expression data by decorrelating GO graph structure. Bioinformatics 22: 1600-1607.
